# Different Structures—Similar Effect: Do Substituted 5-(4-Methoxyphenyl)-1*H*-indoles and 5-(4-Methoxyphenyl)-1*H*-imidazoles Represent a Common Pharmacophore for Substrate Selective Inhibition of Linoleate Oxygenase Activity of ALOX15?

**DOI:** 10.3390/molecules28145418

**Published:** 2023-07-14

**Authors:** Alexander Zhuravlev, Alejandro Cruz, Vladislav Aksenov, Alexey Golovanov, José M. Lluch, Hartmut Kuhn, Àngels González-Lafont, Igor Ivanov

**Affiliations:** 1 Lomonosov Institute of Fine Chemical Technologies, MIREA—Russian Technological University, Vernadskogo pr. 86, 119571 Moscow, Russia; alekszhur95@yandex.ru (A.Z.); aksenov.v.v@edu.mirea.ru (V.A.); golovanov_a@mirea.ru (A.G.); 2 Departament de Química, Universitat Autònoma de Barcelona, Bellaterra, 08193 Barcelona, Spain; alejandro.cruz@uab.cat (A.C.); josemaria.lluch@uab.cat (J.M.L.); angels.gonzalez@uab.cat (À.G.-L.); 3 Shemyakin-Ovchinnikov Institute of Bioorganic Chemistry, Miklihio-Maklaja Str., 16/10c4, 117997 Moscow, Russia; 4 Institut de Biotecnologia i de Biomedicina (IBB), Universitat Autònoma de Barcelona, Bellaterra, 08193 Barcelona, Spain; 5 Department of Biochemistry, Charite—University Medicine Berlin, Corporate Member of Free University Berlin and Humboldt University Berlin, Charitéplatz 1, D-10117 Berlin, Germany; hartmut.kuehn@charite.de

**Keywords:** eicosanoids, lipoxygenase inhibitors, protein–protein interactions, allosteric inhibition, molecular dynamics

## Abstract

Mammalian 15-lipoxygenases (ALOX15) are lipid peroxidizing enzymes that exhibit variable functionality in different cancer and inflammation models. The pathophysiological role of linoleic acid- and arachidonic acid-derived ALOX15 metabolites rendered this enzyme a target for pharmacological research. Several indole and imidazole derivatives inhibit the catalytic activity of rabbit ALOX15 in a substrate-specific manner, but the molecular basis for this allosteric inhibition remains unclear. Here, we attempt to define a common pharmacophore, which is critical for this allosteric inhibition. We found that substituted imidazoles induce weaker inhibitory effects when compared with the indole derivatives. In silico docking studies and molecular dynamics simulations using a dimeric allosteric enzyme model, in which the inhibitor occupies the substrate-binding pocket of one monomer, whereas the substrate fatty acid is bound at the catalytic center of another monomer within the ALOX15 dimer, indicated that chemical modification of the core pharmacophore alters the enzyme–inhibitor interactions, inducing a reduced inhibitory potency. In our dimeric ALOX15 model, the structural differences induced by inhibitor binding are translated to the hydrophobic dimerization cluster and affect the structures of enzyme–substrate complexes. These data are of particular importance since substrate-specific inhibition may contribute to elucidation of the putative roles of ALOX15 metabolites derived from different polyunsaturated fatty acids in mammalian pathophysiology.

## 1. Introduction

Mammalian 15-Lipoxygenases (ALOX15) are lipid peroxidizing enzymes that have been implicated in cell differentiation [1,2,3], in atherogenesis [4,5], in ferroptosis [6,7], in insulin resistance and in adipose tissue inflammation [8,9,10]. These enzymes are able to oxygenate not only free polyunsaturated fatty acids but also complex lipid–protein assemblies such as biomembranes and lipoproteins [11]. ALOX15-derived metabolites of free polyenoic fatty acids (PUFAs) have previously been identified as PPAR-γ ligands with strong antiproliferative activity [12]. The anti-inflammatory role of such metabolites has been also reported [13]. However, in different cancer types and in various inflammation models, ALOX15 and its PUFA metabolites exhibit dual functionality. For instance, ALOX15-derived linoleic acid (LA) metabolites may have a protumorigenic effect in glioblastoma cells [14]. 13(S)-HODE, which is the major ALOX15 metabolite of LA, induced suppression of PPARγ and stimulated tumor growth in prostate cancer via activation of MAP kinase pathway [15]. Lipoxins (LXs) are arachidonic acid (AA)-derived metabolites biosynthesized via the combined activity of different ALOX isoforms, exhibiting strong anti-inflammatory properties [16] and contributing to efferocytosis [17]. 15-HETE and 13-HODE have been found to be new pathogenic effectors of HCMV congenital infection [18]. The pathophysiological role of diverse LA- and AA-derived ALOX15 metabolites made this enzyme a promising target for pharmacological research. Among the number of ALOX15 inhibitors that have been developed so far [19,20,21,22,23,24,25,26], there are only few examples of compounds for which substrate-specific inhibition of LA oxygenation has been described [26,27]. This data are of particular importance since substrate-specific inhibition may contribute to elucidation of the putative roles of ALOX15 metabolites derived from different polyunsaturated fatty acids in mammalian pathophysiology.

The preferential inhibition of oxygenation of one fatty acid over others can hardly be explained by the conventional mechanism of ALOX catalysis, suggesting allosteric regulation of enzyme activity. The crystal structure rabbit ALOX15 (PDB entry 2P0M) [28] contains a mixed protein dimer, in which two structurally different monomers A and B are noncovalently linked with each other via their α2 and α18 helices. In aqueous solution, when the protein motional flexibility is less limited, we observed a monomer–dimer equilibrium for the ligand-free enzyme [29], and these data suggest that rabbit ALOX15 may be present in two conformational states, which differ from each other by the localization of their α2 helices. This hypothesis was supported by small angle X-ray scattering assays [29] and by polyacrylamide gel electrophoresis under native conditions [30]. Similar conformational differences were recently observed in human ALOX12 dimer using cryo-electron microscopy [31]. Site-directed mutagenesis of key amino acids that contribute to ALOX15 inter–monomer interaction, stability assays, molecular dynamics (MD) simulations and kinetic modelling allowed extending conventional model of lipoxygenase catalysis by the following elements: (i) in aqueous solutions, ALOX15 may be present as transient dimers; (ii) effectors (inhibitors or activators) may be bound at the substrate-binding pocket of one monomer and may induce conformational changes in the structure of other monomer that bind fatty acid substrates; and (iii) the alterations in the structure of the catalytic monomer modify the mode of substrate alignment at the active site and, thus, modulate the catalytic activity of the enzyme [27,30].

**Figure 1:**
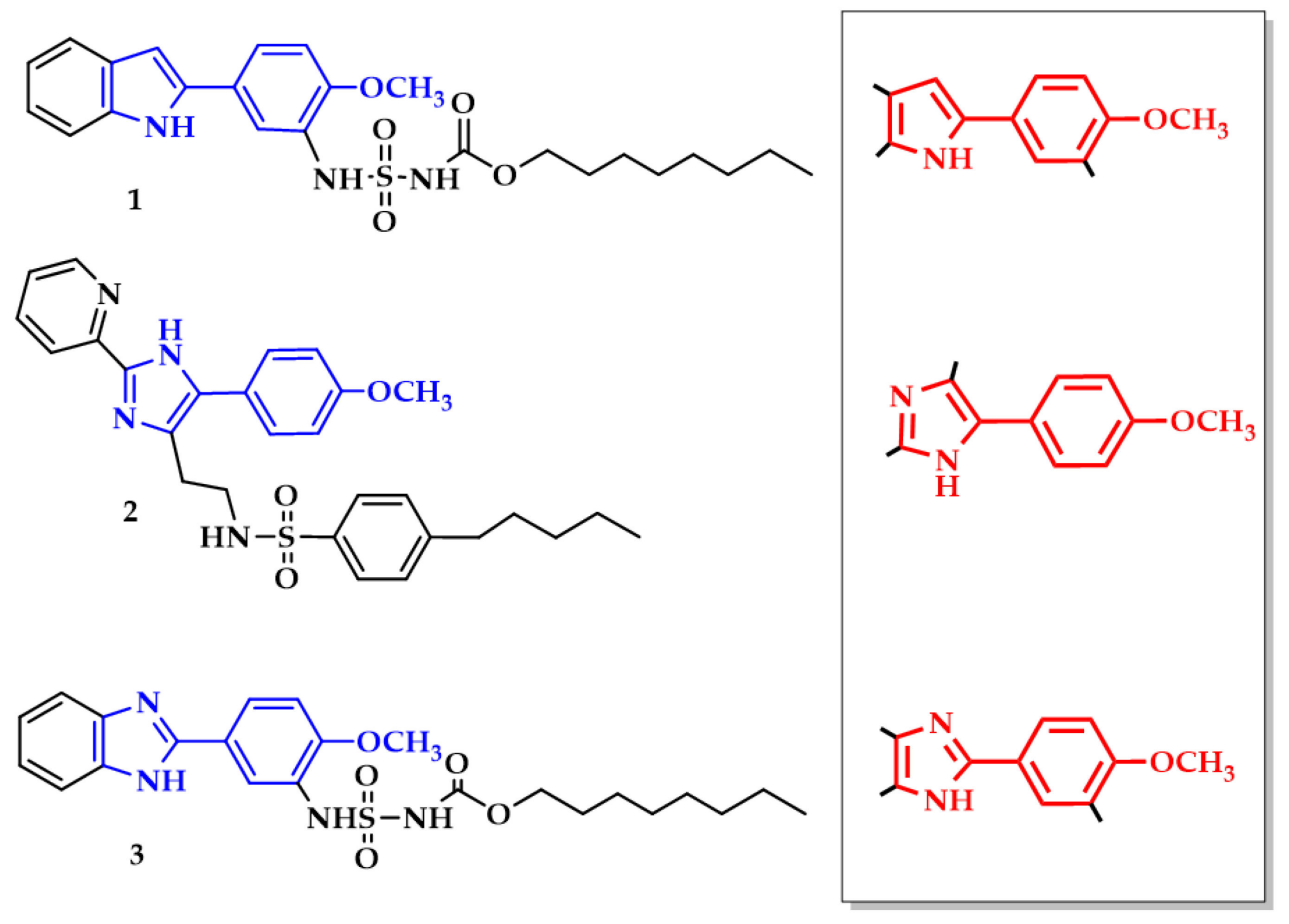
Selected structures of substrate-specific ALOX15 inhibitors to study their structure–activity relationship. (**Left panel**): Detailed structures of selected compounds, (**Right panel**): Common pharmacophore.

In a previous study, we found that the 2-arylindole scaffold **1** with its MeO-group attached in the paraposition of the benzene ring (Figure 1) represents an “allosteric determinant” required for selective inhibition of the linoleate oxygenase activity of ALOX15 [27]. In contrast, the ester group of the sulphocarbamate moiety may be considered an “affinity determinant” required for effective binding of the inhibitor at the enzyme. Like indoles, imidazoles have become important scaffolds for the development of new drugs [32,33]. Thus, imidazole-derived pentylbenzenesulfonamide **2** exhibited a similar effect on rabbit ALOX15 as compound **1** [21]. The selective inhibition of the linoleate oxygenase activity of ALOX15 by both compounds **1** and **2** may be related to their structural similarities (Figure 1).

To explore which structural elements present in compounds **1** and **2** may be critical for allosteric enzyme inhibition, we prepared 4-methoxyphenyl-1*H*-indole- and 4-methoxyphenyl-1*H*-imidazol containing ALOX15 inhibitors **1** and **2**. Next, we added an additional nitrogen atom to the structure of the core pharmacophore of inhibitor **1** to obtain the new 5-(4-methoxyphenyl)-1*H*-benzimidazole derivative **3**. Finally, we compared the inhibitory potencies of compounds **1**–**3** against pure recombinant rabbit ALOX15. To explore the consequences of inhibitor structure on fatty acids alignment at the active site, we performed in silico docking studies and molecular dynamics (MD) simulations using an allosteric enzyme model, in which the inhibitor occupies the substrate-binding pocket of monomer A, whereas monomer B binds the substrate fatty acid at its catalytic center.

## 2. Results

### 2.1. Chemical Synthesis of Target Compounds

To test the validity of our hypothesis, the octyl (N-(5-(1*H*-indol-2-yl)-2-methoxyphenyl)sulfamoyl)carbamate (**1**) was prepared according to the procedure we described previously [27]. N-(2-(5-(4-methoxyphenyl)-2-(pyridin-2-yl)-1*H*-imidazol-4-yl)ethyl)-4-pentylbenzenesulfonamide (**2**) was synthesized from 4-chloro-1-(4-methoxyphenyl)butan-1-one (**4**) according to Weinstein et al. [21], following a slightly modified synthetic protocol (see Supporting Information).

**Scheme 1:**
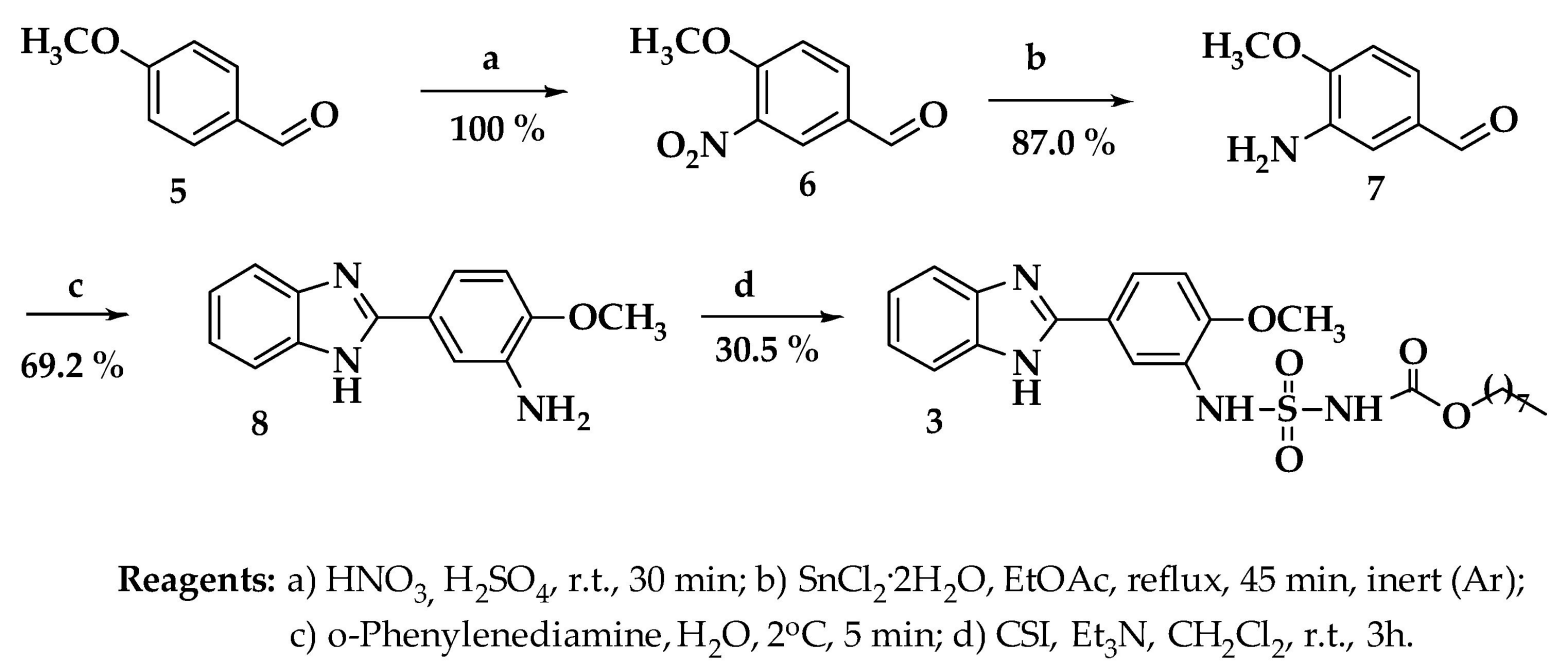
Synthesis of octyl (N-(5-(1*H*-benzo[d]imidazol-2-yl)-2-methoxyphenyl)sulfamoyl)carbamate (**3**).

Finally, the octyl (N-(5-(1*H*-benzo[d]imidazol-2-yl)-2-methoxyphenyl)sulfamoyl)carbamate (**3**) was prepared from the anisaldehyde **5** (Scheme 1). Nitration of aldehyde **5** resulted in compound **6** with an almost 100% yield. For reduction of the nitro group of **6** to the corresponding amine **7** tin (II) hydrochloride was selected due to its high reducing efficiency and the simplicity of the reaction performance. Generally, 2-substituted benzimidazoles can be prepared by condensation reaction between o-phenylenediamines and various aromatic aldehydes that require rather hard autooxidation conditions or the presence of different catalysts [34,35,36,37]. In turn, the reaction of o-phenylenediamine with 3-amino-4-methoxybenzaldehyde (**7**) yielded 70% of the cyclization product **8** when reaction was performed in water at room temperature. The benzimidazole **7** was subsequently transformed to the corresponding sulfamoylcarbamate **3** by the reaction with chlorosulfonyl isocyanate and octanol-1 in the presence of triethylamine [23]. Prior to use, the compounds **1**–**3** were purified by preparative RP-HPLC to reach a ≥98% degree of purity.

### 2.2. Inhibitory Potency of Substituted 4-Methoxyphenyl-1H-indole- and 4-Methoxyphenyl-1H-imidazol-Based Inhibitors ***1**–**3***

**Figure 2:**
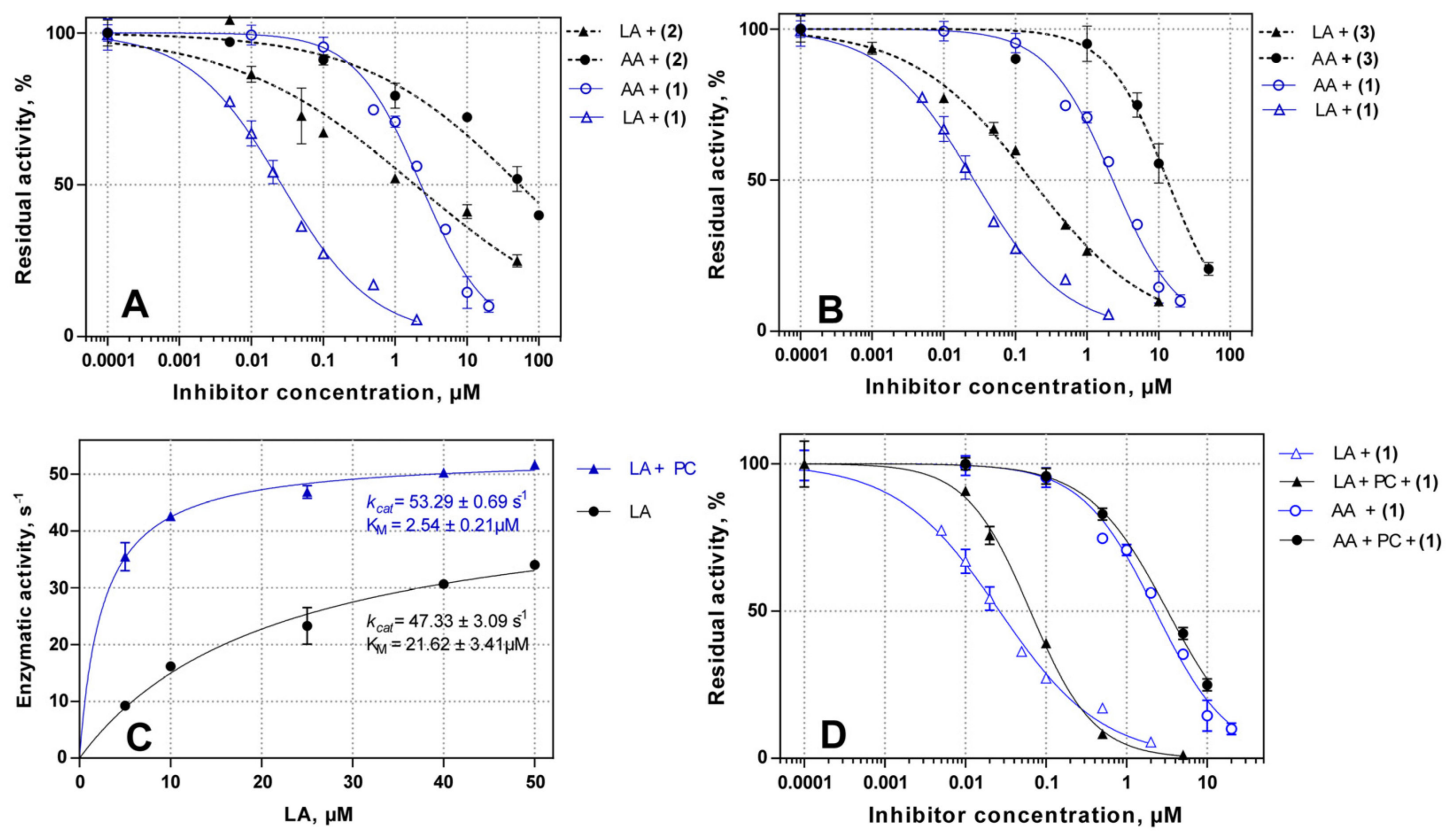
Dose response curves for inhibition of LA- and AA-oxygenase activities of pure recombinant rabbit ALOX15 (**A**,**B**). The LA- and AA-oxygenase activities were assayed in the presence or absence of compounds **1**–**3**. Spectrophotometric assays were performed as described in the Experimental Section and half-maximal inhibitory concentrations (IC_50_) were determined. (**C**) Effect of liposomes (15 µM PC liposomes final concentration) on maximal enzymatic activity (*k_cat_*) and the substrate affinity of rabbit ALOX15. Spectrophotometric assay was performed as described in the Experimental Section. (**D**) Dose–response curves for inhibition of LA- and AA-oxygenase activities of pure recombinant rabbit ALOX15 by compound **1** in the presence of liposomes (15 µM PC final concentration).

The effectiveness of ALOX15 inhibition by the newly synthesized compounds was quantified employing the standard spectrophotometric activity [38] assay that monitors the rate of conjugated diene formation when AA and LA were used as substrates for the pure recombinant rabbit ALOX15. As expected, a high degree of substrate selectivity against linoleic acid was observed for the compounds **1**–**3** with IC_50_(LA)/IC_50_(AA) ratios varying between 0.010 to 0.032 (Table 1). The IC_50_ values of the compound **2** with both LA and AA were almost two orders of magnitude higher than those obtained for the reference compound **1** (Table 1, Figure 2A). In contrast, a moderate loss of potency was observed for compound **3** (Figure 2B). The slopes of the titration curves for compounds **1** and **3** were similar but the slopes of the curves for compound **2** were much less steep. These data and the determined IC_50_-values suggest that compound **2** is a much weaker inhibitor of the linoleate–oxygenase activity of ALOX15.

### 2.3. Substrate Selective Inhibition of LA-Oxygenase Activity of ALOX15 in the Presence of Liposomes

LA has previously been reported to be the preferred substrate for rabbit ALOX15 [30] and the following kinetic constants were calculated for LA and AA in the present study: *k_cat_*^LA^ = 47.3 ± 3.1 s^−1^, K_M_^LA^ = 21.6 ± 3.4 µM, *k_cat_*^AA^ = 21.9 ± 2.1 s^−1^, and K_M_^AA^ = 13.1 ± 1.9 µM. In fact, catalytic efficiency (*k_cat_*/K_M_) of LA oxygenation overcomes that of AA 1.3 times (2.20 s^−1^µM^−1^ and 1.67 s^−1^µM^−1^ for LA and AA, respectively). Binding of ALOX15 to biomembranes strongly activates the fatty acid oxygenase activity of the enzyme [39]. Using L-α-phosphatidylcholine-based liposomes (PC) as membrane mimetics, we here observed an almost 7-fold increase in the LA affinity of ALOX15. Interestingly, the maximal enzymatic activity remained unaffected (Figure 2C). Similar results were obtained for AA. Taken together, this data suggest that binding to liposomes augmented the catalytic efficiency (*k_cat_*/K_M_) of the enzyme and allosteric mechanisms might be involved.

To test whether the ALOX15-liposome interaction may impact the substrate selectivity of ALOX15 inhibition, we recorded dose–response curves for the inhibition of LA- and AA-oxygenase activities of pure recombinant rabbit ALOX15 in the presence of liposomes (Figure 2D). As expected, the IC_50_ values for inhibition of LA and AA oxygenation were almost unaffected by the liposomes. The IC_50_(LA)/IC_50_(AA) ratio of 0.018 suggested a high substrate selectivity of compound **1** towards inhibition of LA oxygenation (Table 1). Thus, liposome-bound ALOX15 may acquire a distinct protein conformation, but allosteric modulation of its enzymatic function and the substrate-specific inhibition appeared to be independent processes that do not interfere with liposome binding.

### 2.4. Docking and MD Simulations of Enzyme–Inhibitor Complexes

**Figure 3:**
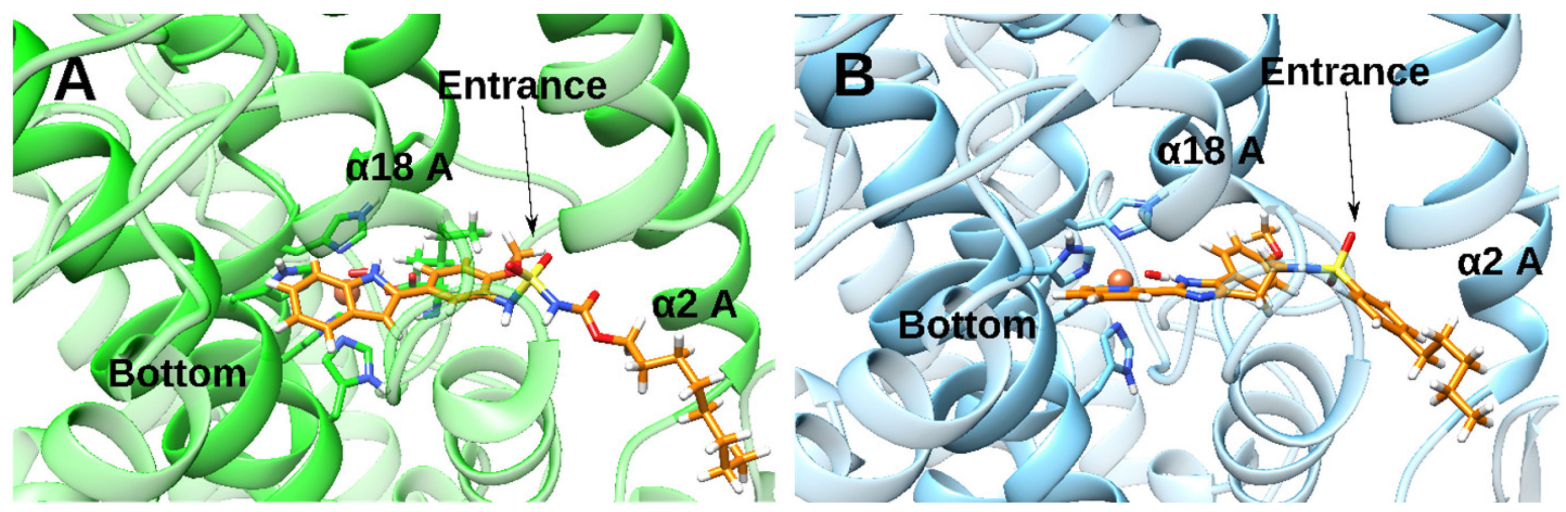
Best-ranking docking pose for compounds **1** (**A**) and **2** (**B**) when their pharmacophore group is bound to the protein pocket of monomer A with the MeO-group located at the cavity side that is defined by helices α2 and α18.

Although the pharmacophore groups of compounds **1** and **2** are structurally different, both compounds show comparable IC_50_(LA)/IC_50_(AA) ratios and this data indicate a similar degree of substrate-specific inhibition. However, compound **2** was a weaker inhibitor as indicated by the higher IC_50_-value and by the slopes of the inhibition curves. To explain this observation, we performed in silico docking calculations of compound **2** inside the binding cavity of monomer A of the dimeric rabbit ALOX15 crystal structure (PDB entry 2P0M) [28]. Due to the bulkiness of compound **2**, no stable docking solution was obtained when this compound was introduced into the substrate-binding pocket of monomer A. This result suggested that the pocket of monomer A needs structural rearrangement to accommodate compound **2** in the substrate-binding pocket. For this reason, additional docking calculations were carried out with compound **2** using the binding cavity of monomer A, once adapted to accommodate compound **1** [27]. In this reorganized structure of the protein, several binding modes of compound **2** were found. However, only two of them involved the tricyclic pharmacophore inside the pocket of monomer A. Finally, the best-ranking poses of those two binding modes were selected. In the first docking mode, the pyridiyl group was located at the bottom of the cavity with its imidazole fragment forming a hydrogen bond with the OH^−^ group of the Fe(III)-OH^−^ cofactor [31]. The methoxyphenyl group of **2** is located at the cavity side (Figure 3B), which is defined by helices α2 and α18, whereas its sulphonamide tail is located close to the entrance of the pocket of monomer A. This binding mode is similar to that of compound **1** (Figure 3A) with its MeO-group located in the same region of the pocket of monomer A. In the second docking binding mode, the pyridyl and methoxyphenyl groups were flipped around. In this case, the methoxyphenyl group is located at the bottom of the cavity, while the pyridyl residue is bound at the part of the cavity defined by helices α2 and α18.

Next, we carried out a 400 ns MD simulation for both enzyme–inhibitor complexes of compound **2** to test the stability of those complexes. Here we found that the two binding modes of compound **2** showed differential behaviour. The first binding mode, in which the methoxyphenyl group is located at the bottom of the cavity, is rather stable along the entire MD simulation period. In this binding mode, the initial blockage of Arg403 and Arg599 in monomer B is released, so that both residues become accessible to the corresponding substrate. The second binding mode of this inhibitor, in which the methoxyphenyl group is located in the part of the cavity that is defined by helices α2 and α18, is also stable, but in this pose the initial blockage of Arg403 and Arg599 is preserved. Therefore, not only the presence of the MeO-group, but also its position inside the binding pocket of monomer A may be important.

Although the binding modes of compounds **1** and **2**, which have the methoxyphenyl group occupying the cavity defined by helices α2 and α18, look similar, the molecular interactions between both compounds and the enzyme may be different (Table 2 and Table 3 and Supporting Information Figure S1). 

For that reason, the degree of blockage of Arg403 and Arg599 is not the same in both cases (Supporting Information Figure S2). From an inhibitory point of view, the blockage of those two arginine residues along with the modification that cavity B undergoes (Supporting Information Figure S3) are the most relevant conformational changes induced by compounds **1** and **2**. In the absence of these inhibitors, the two arginine residues attach to the substrate’s carboxylate so that the shape of cavity B becomes totally different [27,30].

**Figure 4:**
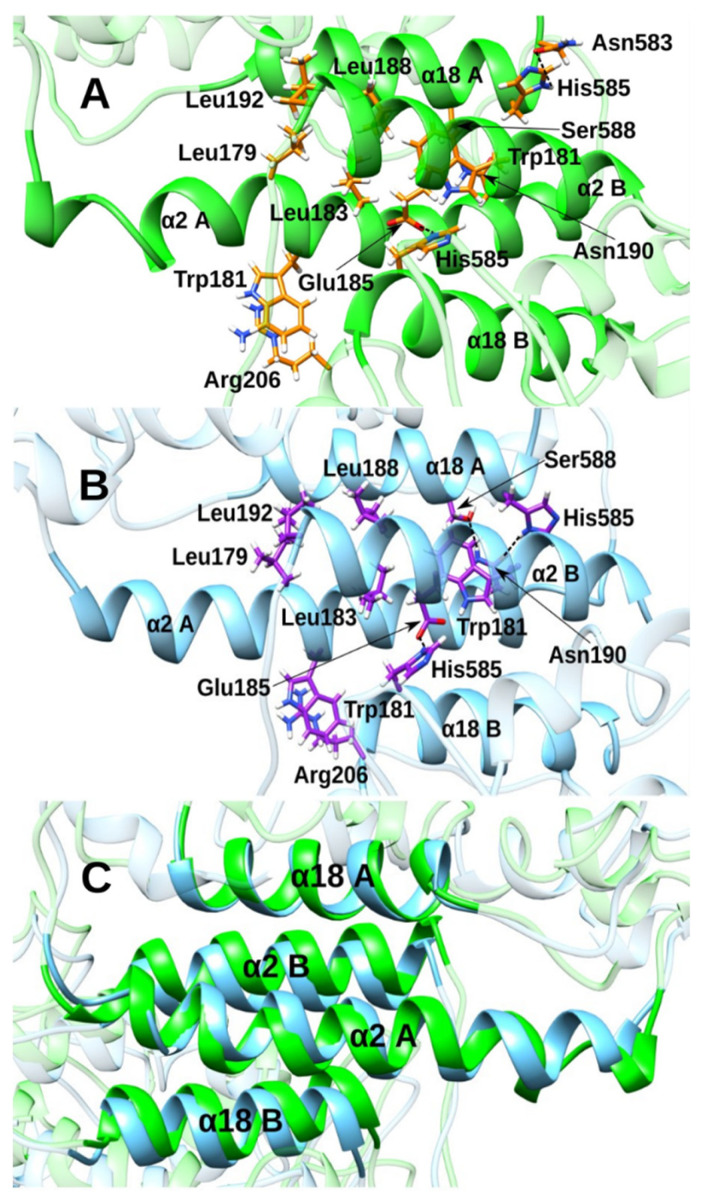
Interface representation from MD simulations of the dimeric rabbit ALOX15 complex when compound **1** (**A**) and compound **2** (**B**) are bound to its monomer A. The structures of compounds **1** and **2** are not shown for clarity. (**C**) An overlap of both inhibitor interfaces.

Both compounds **1** and **2** induced similar effects on the inter-monomer interface, but some subtle differences can be observed (Figure 4A–C). The position of helix α18A and its overall structure is not differentially impacted when either compound **1** or **2** is bound. Helices α2B and α18B also show a similar structure, but the relative positions of these structural elements as a whole did slightly differ in the two complexes. Despite these similarities, we found a major difference for helix α2A. When the indole derivative **1** was bound, the structure of the two first helix loops is modified so that the helix becomes slightly bent. In contrast, after binding of inhibitor **2**, all loops of helix α2 adopt a regular alpha-helical conformation so that the helix as a whole remains unaffected.

It has previously been suggested that a hydrophobic cluster of leucines (Leu 179, Leu 183, Leu 188 and Leu 192) and the side chains of Trp181 and His585 of both monomers within the ALOX15 dimer are crucial for the structural stability of the inter-monomer interface [30,40]. For this reason, the interactions of the aforementioned residues in the presence of inhibitors **1** and **2** were analysed. Here we found that binding of the two compounds interrupts the original Leu zipper conformation (PDB entry 2P0M) of the hydrophobic cluster involved in the inter-monomer interaction. In fact, the side-chains of Leu’s of one monomer are faced with the corresponding side-chains of the counterpart Leu’s on the other monomer. Moreover, the hydrophobic Leu-zipper cluster stabilizing the ALOX15 dimer appears to be more seriously disturbed when compound **1** was bound. With compound **2**, more subtle structural alterations were observed. Summarising these data, one can conclude that both inhibitors modify the structure of the inter-monomer interface between the two monomers within an ALOX15 dimer in different ways.

Finally, we selected the octyl (N-(3-(1*H*-indol-2-yl)phenyl)sulfamoyl)carbamate [27], which does not contain a 4-MeO group at the core of the pharmacophore and, hence, does not block Arg403 and Arg599 [27]. Next, we carried out a new MD simulation with this compound to ensure that the structural changes that were observed when compound **2** was bound at ALOX15 monomer A are not biased and can be reverted. Along the MD simulation with the octyl (N-(3-(1*H*-indol-2-yl)phenyl)sulfamoyl)carbamate, the initial conformational changes induced by compound **2** were reverted to a large extent, since the blockage of Arg403 and Arg599 was released (Supporting Information Figure S2C). Although Arg599 is still a bit obstructed, now both arginine residues are accessible and could attach to the substrate carboxylate group. Therefore, we can conclude that using the enzyme structure adapted to accommodate compound **1** as a starting structure does not suppose any significant bias concerning the allosteric effects observed.

### 2.5. Impact of the Allosteric Inhibitors on Binding of LA and AA along Their MD Simulations

**Figure 5:**
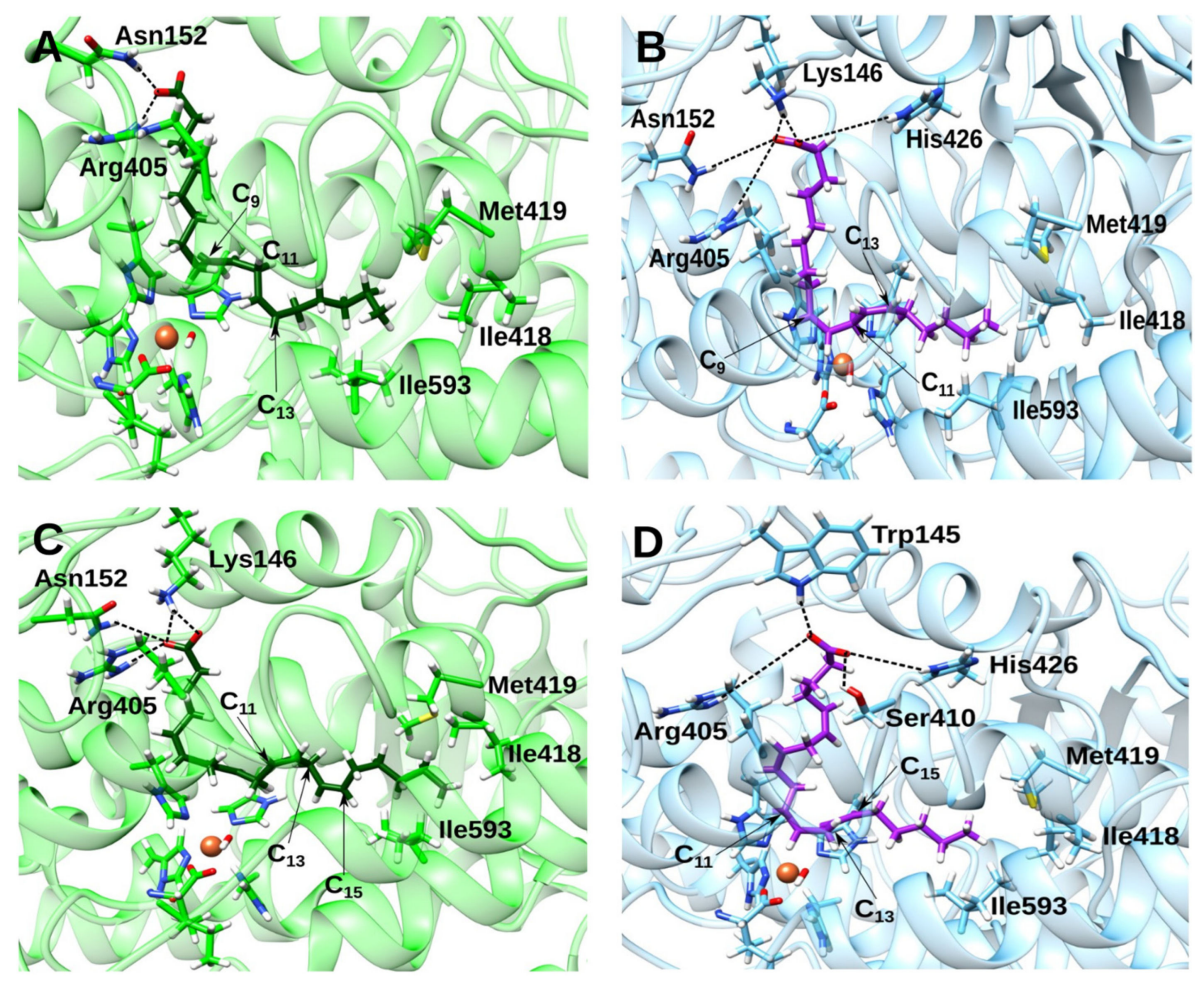
Binding modes of LA in the presence of indole derivative **1** (**A**) and imidazole derivative **2** (**B**) and binding modes of AA in the presence of indole derivative **1** (**C**) and imidazole derivative **2** (**D**).

We have previously reported [27] that the binding mode of LA at the active site of monomer B was altered with compound **1** bound to the pocket of monomer A of the ALOX15 dimer. The substrate carboxylate group forms hydrogen bonds with Arg405 and Asn152 showing a binding mode adapted to the modified cavity (Figure 5A).

When compound **2** is bound to monomer A, LA inside the active site of monomer B adopts a binding position which is similar to that induced by compound **1**. This result is not surprising, since the inhibitor-induced modifications in the structure of the cavity of monomer B by both compounds are similar (Supporting Information Figure S3A,B). A representative structure of the most populated cluster is given in Figure 5B. In this case, the carboxylate hydrogen bonds are mainly established with Lys146 (d(HZ2-O1) = 2.47 Å; d(HZ2-O2) = 1.67 Å), but weaker hydrogen bonds are also formed with Arg405, Asn152, and His426. The tail of linoleic acid remains in a similar position in monomer B, regardless of whether compound **1** or compound **2** is present in the cavity of monomer A. However, when compound **2** is bound in the substrate-binding pocket of monomer A, the LA molecule adopts a more L-shaped conformation in the substrate-binding pocket of monomer B (Figure 5B). C_11_ and the H_11proS_ atom of LA are located closer to the Fe(III)-OH^−^ cofactor so that the number of catalytically well-oriented structures (Table 4) are considerably higher when the imidazole derivative **2** is bound to conformer A (84.80% vs. 34.00%).

Similarly, when compound **2** is bound to monomer A, the binding mode of AA inside the active side of monomer B is similar to that induced by compound **1**. Regarding its carboxylate group, when compound **1** acts as an inhibitor, hydrogen bonds are formed with Lys146, Arg405, and Asn152 (Figure 5C). In contrast, in the presence of compound **2**, the AA carboxylate group mainly interacts with Trp145 (d(NH-O1) = 2.01 Å), and forms weaker hydrogen bonds with Arg405, Ser410, and His426 (Figure 5D). Again, the tail of the substrate fatty acid remains located in the same region, regardless of which inhibitor is present in the cavity of monomer A. However, in the presence of compound **1**, the AA becomes more extended and its C_13_ and H_13proS_ atoms are farther away from the OH^−^ group. Instead, in the presence of compound **2** in monomer A, AA adopts an L-shaped conformation and now, its C_13_ and H_13proS_ atoms become somewhat closer to the OH^−^ group and the percentage of well-oriented structures is higher (93.38% vs. 87.90%) (Table 4). The percentage of well-oriented structures for hydrogen abstraction follows the trend of IC_50_ experimental values for the considered inhibitors.

## 3. Discussion

### 3.1. Liposomes Do Not Affect the Substrate Selective Inhibition of LA-Oxygenase Activity of ALOX15

The spatial orientation of hydrogens attached to the bisallylic methylene groups (C_11_ for LA and C_13_ for AA) relative to the Fe(III)-OH^−^ cofactor is important for the catalytic mechanism of the lipoxygenase reaction [41,42]. The length of the aliphatic hydrocarbon chain, the number of the double bonds, their positions, and their geometry impact the alignment of a fatty acid substrate at the active site of ALOX15 and makes LA a better substrate for the wild-type enzyme when compared to AA [30]. In this study, we found that the catalytic activity (*k_cat_*) calculated for LA oxygenation was two times higher than that of AA with *k_cat_*^LA^ = 47.3 ± 3.1 s^−1^ and *k_cat_*^AA^ = 21.9 ± 2.1 s^−1^ for LA and AA, respectively. In a kinetic assay, PC-based liposomes significantly augment the substrate affinity (decrease in K_M_) but had no effect on *k_cat_* (Figure 2C).

Mammalian ALOX15 are capable of oxygenating not only free polyunsaturated fatty acids but also unsaturated ester lipids even when these substrates are incorporated in lipid–protein assemblies like biomembranes and lipoproteins. It has been reported previously that interaction of ALOX15 with certain types of lipids, predominantly with phosphatidylinositol bisphosphates, results in increased enzyme activity in a Ca^2+^-dependent manner [39], but the molecular basis for this effect has not been clarified. Here we suggest that structural alterations of the enzyme that are induced by the binding of the enzyme to liposomes might be involved in the activation process. A single ALOX15 monomer consists of an N-terminal β-barrel domain (PLAT domain) and a C-terminal catalytic domain and both structural subunits share a 1600-Å^2^ interdomain interface (PDB entry 2P0M). This architecture is important for protein stability and catalytic activity [43]. Several partly solvent-exposed (more than 30%) nonpolar amino acids of the PLAT domain, which are clustered at the interdomain contact plane, may be involved in interaction with the hydrophobic core of membrane phospholipids [44]. In contrast, the compact structure of soybean LOX-1 rule out any major motional flexibility of this enzyme molecule in aqueous solutions [45] and this enzyme does not exhibit any membrane oxygenase activity.

**Figure 6:**
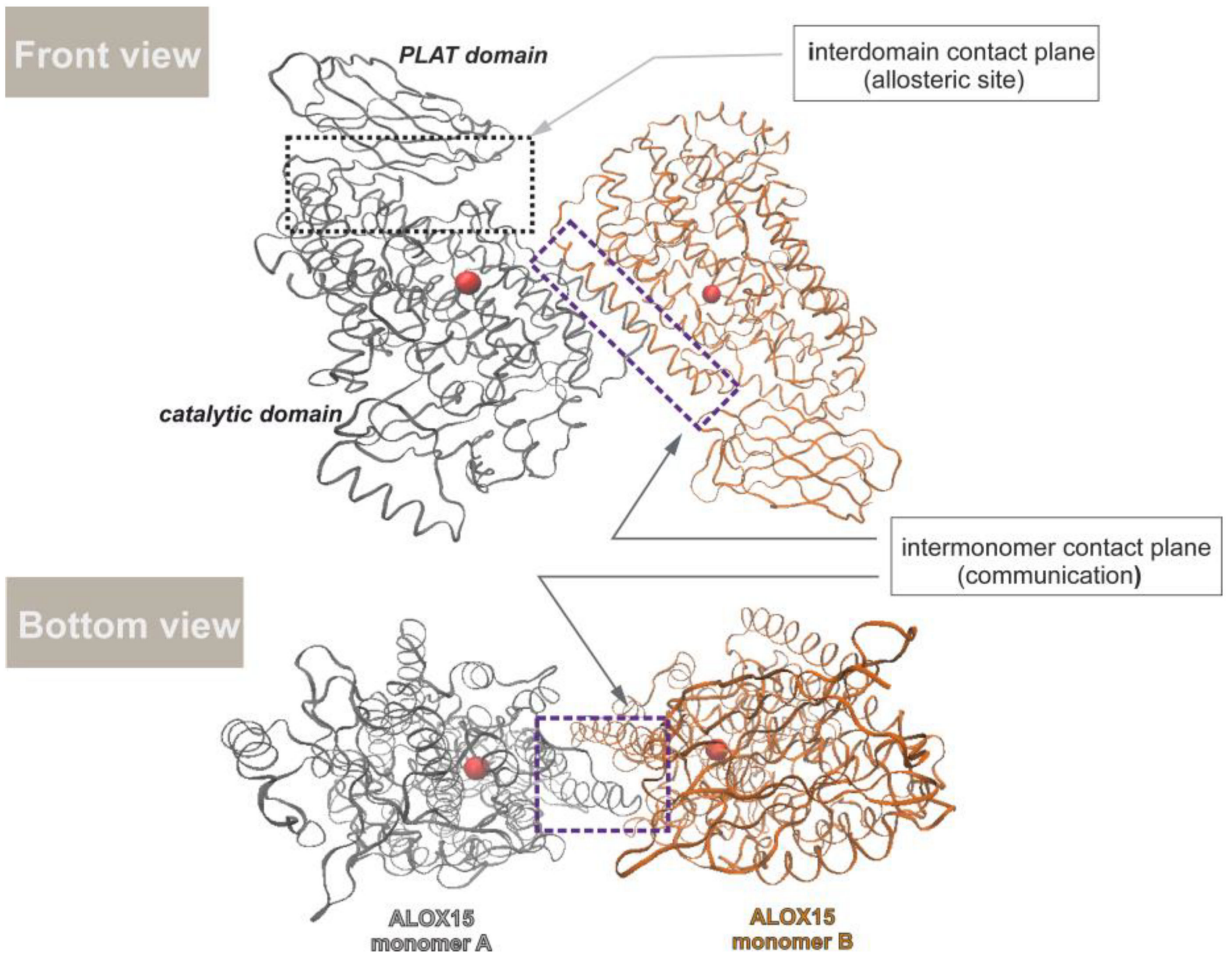
Interdomain allosteric site and intermonomer communication sites within ALOX15 dimer.

3-*O*-acetyl-11-keto-b-boswellic acid (AKBA), which binds the interdomain contact plane may additionally activate cellular ALOX15 via this allosteric site [46]. Hydrophobic burial of AKBA into the interdomain pocket (Figure 6) appears to be the primary molecular determinant for its binding, but other residues that are located close to helix α2 may also be involved in the formation of H-bonds and/or electrostatic interactions. 

The helix α2 of one ALOX15 monomer interacts with the corresponding helix of the partner monomer via hydrophobic interactions and this interdomain network is important for dimerization of ALOX15 and for allosteric behavior of the dimeric enzyme [27]. The structural flexibility of rabbit ALOX15 in aqueous solutions enables a high degree of motional flexibility of regulatory PLAT domain and is a precondition for ALOX15 dimerization [29]. Hence, liposome binding may not only affect the structure of catalytic domain of rabbit ALOX15 but might also impair the inter-monomer interactions within an ALOX15 dimer. Analysis of the dose response curves for inhibition of LA- and AA-oxygenase activities of pure recombinant rabbit ALOX15 in the presence of PC-based liposomes have shown that selectivity of inhibition of LA-oxygenase activity was almost unaffected (Figure 2D). Although no significant impact on enzymatic catalysis of ALOX15 was observed in previous reports with AA or LA incorporated in either PC or PE vehicles [39], differences in the assay system, the absence of calcium and the particles composition may be the reason. In summary, PC-based liposomes increase catalytic efficiency (*k_cat_*/K_M_) of enzymatic catalysis of rabbit ALOX15 via allosteric mechanisms and this activation does not interfere with interdomain communication within an ALOX15 dimer (Figure 6). The substrate-specific inhibition of the LA-oxygenase activity by octyl (N-(5-(1*H*-indol-2-yl)-2-methoxyphenyl)sulfamoyl)carbamate is retained in the presence of liposomes.

### 3.2. Effect of the Inhibitor Structure on Cooperativity of Rabbit ALOX15 Monomers

Compounds **1** and **2** show very small structural fluctuations in the binding cavity of conformer A, and both inhibitors modify the shape of the substrate-binding cavity of monomer B in a similar way (see Supporting Information Figure S3A,B). When compounds **1** and **2** bind to monomer A of the ALOX15 dimer, they differentially modify the interactions of key amino acid residues that are involved in ALOX15 dimerization. Previous mutagenesis studies combined with SAXS measurements [40] suggested the importance of a hydrophobic Leu-zipper cluster and the Trp181/His585 interaction (Trp181 belongs to the α2 helix of one monomer and His585 to the α18 helix of the other one) for ALOX15 dimerization and the catalytic properties of the ALOX15 dimer. When compounds **1** and **2** act as inhibitors, the electron cloud of Trp181 of the conformer A [Trp181(A)] interacts via coulomb forces with the side chain of Arg206(B) (Figure 4A,B). In addition, His585(A) forms a hydrogen bond with different partners, but the identity of the partner residues is variable. When compound **1** acts as an inhibitor (Figure 4A), a hydrogen bond is established with the sidechain keto group of Asn583 (A) (d(His585-HD1-OD1-Asn583) = 2.911 Å); however, this hydrogen bond has poor directionality. In contrast, when compound **2** acts as inhibitor (Figure 4B), a hydrogen bond is formed with the sidechain keto group of Asn190 (A) (d(His585-HD1-OD1-Asn190) = 1.962 Å) and this bond has a stronger directionality. Trp181 (monomer B) is another important element for the inter-monomer interaction. When compound **1** acts as an inhibitor (Figure 4A), the electron cloud of its sidechain ring interacts via coulombic forces with the sidechain OH group of Ser588 (A) and the sidechain NH_2_-group of Asn190 (A). In contrast, when compound **2** acts as an inhibitor (Figure 4B), Trp181(B) forms a hydrogen bond with the sidechain carboxylate group of Glu185 (B) (d(Trp181-HE1-OE1-Glu185) = 2.115 Å). In addition, the electron cloud of its sidechain ring interacts with the sidechain NH_2_-group of Asn190 (A), which in turn forms a hydrogen bond with the sidechain OH group of Ser588 (A) (d(Asn190-HD21-OG-Ser588) = 1.984 Å). Finally, His585 (B) forms a hydrogen bond with the sidechain carboxylic group of Glu185 (B), regardless of whether compound **1** or **2** is bound in the substrate-binding pocket of the monomer A. However, the strength of this interaction may be different (for compound **1**, d(His585-HD1-OE1-Glu185) = 2.065 Å; for compound **2**, d(His585-HD1-OE1-Glu185) = 1.943 Å). These differences may be responsible for the differential allosteric effects. In fact, the interactions observed when indole **1** is bound at the cavity of monomer A induced a deformation of the first two loops of helix α2A, which is not the case for compound **2** binding. These structural alterations are further translated to the cavity of the other monomer, which accommodates the fatty acid substrate. In fact, in the case of compound **2** binding the substrate molecules adopt L-shaped conformations, whereas in the presence of compound **1** the substrate molecules become more extended.

**Figure 7:**
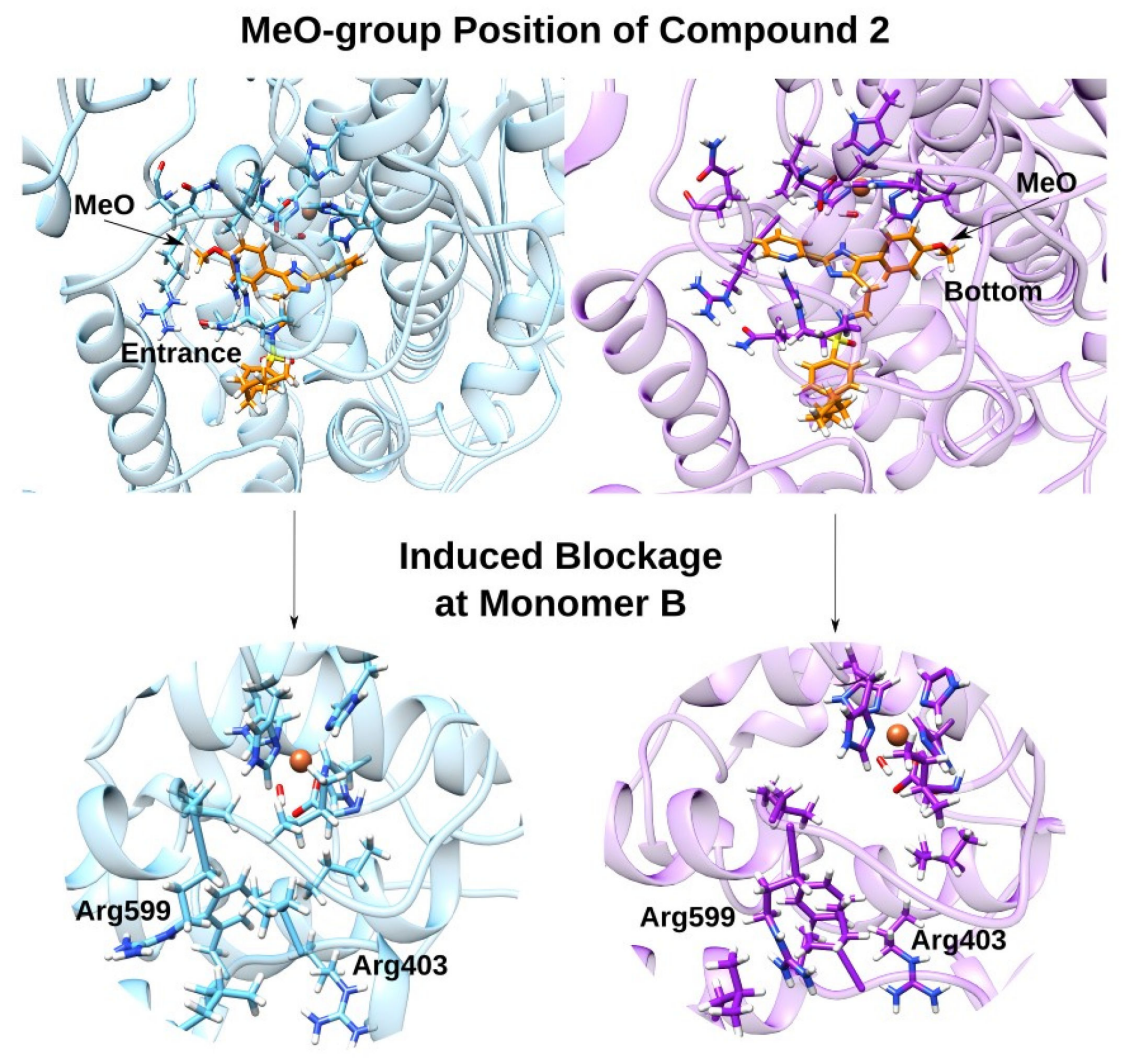
Effect of the MeO-group of compound **2** on allosteric inhibition of ALOX15. On the upper left, compound **2** binding mode with the MeO-group located at the cavity entrance (like compound **1**) correlates with a blockage of Arg599 and Arg403 (plot on the lower left). On the upper right, compound **2** binding mode with the MeO-group located at the cavity bottom correlates with more accessible Arg599 and Arg403 residues like in the absence of inhibitor (plot on the lower right).

Moreover, our results show that allosteric effects not only depend on the presence of the MeO-group in the inhibitor’s structure but also on its location inside the binding pocket of monomer A. The MeO-group position correlates with the degree of blockage of the two Arg residues in monomer B responsible for the attachment of the substrate’s carboxylate group in the absence of inhibitor. This conclusion is summarized in Figure 7.

## 4. Materials and Methods

### 4.1. Chemistry

The solvent and reagents were purchased from Acros (Geel, Belgium) or Sigma-Aldrich (Schnelldorf, Germany) and were used without further purification unless otherwise noted. ^1^H and ^13^C NMR spectra were recorded with a 300 MHz Brucker MSL spectrometer in CDCl_3_, acetone-d_6_ or CD_3_OD using tetramethylsilane as the internal standard for ^1^H-NMR. Chemical shifts in ^13^C-NMR were referenced to the residual carbon signal of CDCl_3_, acetone-d_6_ or CD_3_OD at δ ^13^C = 77.19, 206.26 ppm or 49.00, respectively. Chemical shifts are given in ppm, spin–spin interaction constants in Hz. Flash column chromatography was carried out using silica gel (Acros, Germany, particle size 60–200 μm) as stationary phase. Silica gel 60 F^254^ plates (Merck, Germany) were used for thin-layer chromatography (TLC). Compounds were detected under UV light or after staining with an ethanolic (3%) solution of vanillin. Analytical HPLC of all compounds was performed on a Shimadzu LC-10Avp liquid chromatograph equipped with an SPD-10Advp UV detector (Japan) using different C18 columns and different mobile phase compositions at a flow rate of 1 mL/min. Preparative HPLC was performed using a Knauer HPLC pump 64 system coupled with a differential refractometer and UV-VIS detector (Knauer, Germany). Isocratic elution systems of different compositions of solvents A and B at a flow rate of 25 mL/min were applied to achieve the best chromatographic performance. Mass spectra (EI) were recorded on an Agilent a 6890N gas chromatograph coupled with 5973N mass spectral detector (Agilent, Santa Clara, CA, USA) using a DB-5ms column (30 m, coating thickness 0.5 μm, Agilent J&W, Palo Alto, CA, USA). An injector temperature of 250 °C, an ion source temperature of 230 °C and the electron energy of 70 eV were set. Helium was used as carrier gas at a flow rate of 1 mL/min. Samples were eluted using the following temperature program: isothermally at 70 °C for 5 min, then from 70 to 290 °C at a rate of 30 °C/min, followed by isothermal step at 290 °C for 10 min. Finally, the column was conditioned at 310 °C for 30 min. Mass spectra (ESI) were recorded on Agilent 6160 (Agilent, Singapore) either in the positive or negative ionization mode. The mass spectrometer and source parameters were set up as follows: capillary voltage 3.5 kV and 4 kV for positive and negative ionization, respectively; source temperature 65 °C; desolvation temperature 350 °C; and flow rate of desolvation gas 600 L/h.

*4-Methoxy-3-nitrobenzaldehyde* (**6**). To a solution of 4-methoxybenzaldehyde **5** (2 g, 14.69 mmol) in 7.32 mL of H_2_SO_4_, a mixture of 1 mL of HNO_3_ and 1.47 mL of H_2_SO_4_ was added dropwise at 0 °C. The resulting mixture was kept for 30 min at room temperature, then poured onto ice and organic products were extracted with CH_2_Cl_2_ (4 × 25 mL). The combined organic extracts were dried over Na_2_SO_4_ and concentrated under the reduced pressure. The raw product was purified by column chromatography on silica gel using isocratic eluent system (Pet/EtOAc, 1:1 by vol.). Yield: 2.65 g (99.7%). R_f_ = 0.36 (Pet/EtOAc, 1:1, by vol.). ^1^H NMR (300 MHz, CDCl_3_) δ = 9.93 (s, 1H), 8.33 (d, *J* = 2.1 Hz, 1H), 8.09 (dd, *J* = 8.7, 2.1 Hz, 1H), 7.26 (d, *J* = 8.7 Hz, 1H), 4.07 (s, 3H). ^13^C NMR (75 MHz, CDCl_3_) δ = 57.18, 113.95, 127.34, 129.05, 134.96, 139.86, 157.21, 188.91. MS (EI, 70 eV) *m*/*z* (%): 181 (79), 151 (48), 134 (100), 119 (98), 105 (56), 77 (91).

*3-Amino-4-methoxybenzaldehyde* (**7**). SnCl_2_·2H_2_O (16.61 g, 73.48 mmol) was added to a solution of 4-methoxy-3-nitrobenzaldehyde **6** (2.66 g; 14.69 mmol) in 30 mL of EtOAc and the mixture was refluxed for 30 min under Ar atmosphere. The reaction mixture was then quenched with ice and NH_3_·H_2_O was added to reach pH 7–8. Organic products were extracted with EtOAc (7 × 100 mL), and the combined organic phase was washed with saturated solution of NaCl (150 mL), dried over Na_2_SO_4_ and concentrated under the reduced pressure. The raw product was purified by column chromatography on silica gel using isocratic eluent system (Pet/EtOAc, 1:1, by vol.). Yield: 1.93 g (87.0%). R_f_ = 0.35 (Pet/EtOAc, 1:1, by vol.). ^1^H NMR (300 MHz, CDCl_3_) δ = 9.78 (s, 1H), 7.26 (dd, *J* = 8.1, 2.0 Hz, 1H), 7.22 (d, *J* = 2.0 Hz, 1H), 6.87 (d, *J* = 8.1 Hz, 1H), 3.92 (s, 3H). ^13^C NMR (75 MHz, CDCl_3_) δ = 55.79, 109.62, 112.99, 123.72, 130.33, 136.99, 152.42, 191.55. MS (EI 70 eV) *m*/*z* (%): 151 (100), 136 (77), 108 (24), 80 (57).

*5-(1H-Benzo[d]imidazol-2-yl)-2-methoxyaniline* (**8**). 1,2-diaminobenzene (866 mg, 7.947 mmol) was added to a precooled 1–2 °C solution of 3-amino-4-methoxybenzaldehyde (300 mg; 1.987 mmol) in 15 mL of H_2_O and the mixture was kept for 30 min at room temperature. Organic products were extracted with CH_2_Cl_2_ (4 × 50 mL). The combined organic extracts were dried over Na_2_SO_4_ and concentrated under reduced pressure. The product was purified on silica gel using an isocratic eluent system (Pet/EtOAc, 3:2, by vol.) followed by preparative HPLC on Luna C18 (75 × 30 мм, 5 µm) in MeOH/H_2_O/NH_3_·H_2_O (60:40:0.5%, by vol.). Yield: 328 mg (69.2%). R_f_ = 0.45 (Pet/EtOAc, 1:2, by vol.). Analytical HPLC: R_t_ = 2.94 min (254 and 310 nm), Kinetex F5 (100 × 3 mm, 2.6 µm) MeOH/H_2_O/NH_3_·H_2_O (60:40:0.5%, by vol.). ^1^H NMR (300 MHz, acetone-d_6_) δ = 7.62 (d, *J* = 2.1 Hz, 1H), 7.53 (dd, *J* = 6.0, 3.2 Hz, 2H), 7.47 (dd, *J* = 8.3, 2.2 Hz, 1H), 7.15 (dd, *J* = 6.0, 3.2 Hz, 2H), 6.92 (d, *J* = 8.4 Hz, 1H), 3.88 (s, 3H). ^13^C NMR (75 MHz, acetone-d_6_) δ = 55.92, 111.09, 113.13, 116.56, 122.57, 124.23, 138.74, 149.43, 153.24. MS (ESI): [M + H]^+^ = 240.20.

*Octyl (N-(5-(1H-benzo[d]imidazol-2-yl)-2-methoxyphenyl)sulfamoyl)carbamate* (**3**). To a solution of chlorosulfonyl isocyanate (342 µL, 3.912 mmol) and octanol (445 µL, 2.817 mmol), 3 mL of dry CH_2_Cl_2_ Et_3_N (587 µL, 4.225 mmol) was added, and the mixture was stirred for 15 min at rt. After that, a solution of 5-(1H-benzo[d]imidazol-2-yl)-2-methoxyaniline (374 mg; 1.565 mmol) (**8**) in 3 mL of dry CH_2_Cl_2_ was added to the mixture and the resulting mixture was kept for 3h at room temperature. After the reaction was complete, it was quenched with water (2 mL). The organic layer was separated and dried over Na_2_SO_4_, concentrated under reduced pressure, and the raw product was purified on silica gel using in CH_2_Cl_2_/MeOH (40:1, by vol.) followed by preparative HPLC on Silasorb 600 C18 column (250 × 10 мм, 10 µm) and CH_2_Cl_2_/2-propanol (30:1, by vol.) as a mobile phase. Yield 654 mg (88.2%). R_f_ = 0.40 (THF/Pet, 3:2, by vol.). Analytical HPLC: Rt = 9.91 min (254 and 310 nm), Nucleosil 100–7 C18 column (250 × 4 mm, 7 µm) in CH_2_Cl_2_/2-propanol (30:1, by vol.). ^1^H NMR (300 MHz, CD_3_OD) δ = 8.16 (d, *J* = 2.1 Hz, 1H), 7.82 (dd, *J* = 8.6, 2.2 Hz, 1H), 7.59 (dd, *J* = 6.1, 3.2 Hz, 2H), 7.27 (dd, *J* = 6.1, 3.2 Hz, 2H), 7.17 (d, *J* = 8.6 Hz, 1H), 4.04 (t, *J* = 6.5 Hz, 2H), 3.94 (s, 3H), 1.50 (p, *J* = 6.9 Hz, 2H), 1.18–1.11 (m, 10H), 0.81 (t, *J* = 6.6 Hz, 3H). ^13^C NMR (75 MHz, CD_3_OD) δ = 14.39, 23.63, 26.76, 29.70, 30.24, 30.26, 32.88, 56.80, 67.24, 112.59, 115.59, 120.42, 122.98, 124.15, 124.99, 128.52, 139.47, 152.83, 153.40, 154.07. MS (ESI): [M + H]^+^ = 475.20.

### 4.2. Biochemistry and Biotechnology

The chemicals used were obtained from the following sources: arachidonic acid (5Z,8Z,11Z,14Z-eicosatetraenoic acid) and linoleic acids from Cayman Chem (distributed by Biomol, Hamburg, Germany), HPLC grade methanol, acetonitril and acetic acid from AplyChem (Darmstadt, Germany), isopropyl-β-d-thiogalactopyranoside (IPTG) from Molecula (Munich, Germany), and L-a-Phosphatidylcholine from Avanti Polar Lipids (Alabaster, AL, USA). The *E. coli* strain BL21(DE3)pLysS were purchased from Invitrogen (Carlsbad, CA, USA). Peptone medium was obtained from Greenvan (Moscow, Russia), glucose from Biotech Rosva (Kaluga, Russia), antifoam «Sofexil-1520» from Sofex silicone (Moscow, Russia), ampicillin from ApliChem (Darmstadt, Germany) and thiamine hydrochloride from Sigma-Aldrich (Darmstadt, Germany). Western-blotting was performed with anti-His-tag antibodies-HRP from Sigma-Aldrich (Darmstadt, Germany). Protein mass marker PageRuler™ Prestained was purchased from Thermo Fisher Scientific (Waltham, MA, USA).

#### 4.2.1. Expression of Rabbit ALOX15 in Bioreactor

Wild-type rabbit ALOX15 was expressed as N-terminal His-tag fusion proteins in *E. coli* using the pET-15b prokaryotic expression plasmid. The transformed cells of *E. coli* BL21 (DE3) pLysS/pET-15b/rabbitALOX15/AMPr were stored with 15% glycerol in cryovials at −70 °C. An inoculum medium contained 5.0 g/L glucose, 15.0 g/L complex peptone medium and 10.0 g/L NaCl. The starting culture (300 mL) was generated by incubation for 12 h at 32 °C and 180 rpm. The main culture was grown in a 30 L fermenter (MBR Bioreactor AG, Wetzikon, Switzerland) with a 10 L working volume operated in fed-batch mode. Temperatures for cultivation and induction were maintained at 37 °C and 30 °C, respectively. A pH value of 7.0 was controlled by consequent addition either of 25% (*v*/*v*) ammonium hydroxide or 30% (*v*/*v*) orthophosphoric acid solutions. The aeration rate was set to 1 VVM. Induction of the expression of the recombinant enzyme was performed at OD_600_ = 2.3 by the addition of 0.238 g/L IPTG. The growth medium contained the following ingredients (in grammes per L): 2.0 g/L glucose; 61.0 g/L complex peptone medium; 5 g/L NaCl; 0.1 g/L antifoam, 0.1 g/L and 0.05 g/L ampicillin before the cultivation and induction, respectively; 0.5 g/L magnesium sulfate heptahydrate (MgSO_4_·7H_2_O); 0.2 g/L thiamine hydrochloride; and 6.0 g/L dibasic potassium phosphate trihydrate (K_2_HPO_4_ × 3H_2_O). Bacteria were harvested by centrifugation using Thermo Scientific Sorvall LYNX 6000 (Thermo Fisher Scientific). On average, each fermentation yielded 300 g biomass.

#### 4.2.2. Enzyme Purification

The bacterial pellet (40 g) was reconstituted in either 50 mL PBS and cells were lysed with Branson Sonifier 250 (Branson Ultrasonics, Fremont, MA, USA), cell debris was spun down for 30 min at 13,000 rpm and 4 °C and the lysis supernatant was used for further protein purification. For this purpose, 10–30 bacterial lysis supernatant was added to 0.3 mL of Ni-Agarose bed (Macherey-Nagel, Düren, Germany). This mixture was incubated for 1h at 4 °C, centrifuged for 10 min (1700 rpm), and the pellet was transferred to an open bed column. The column was washed with 50 mM phosphate buffer containing 300 mM NaCl (pH 8.0) and adhering proteins were eluted with the same buffer containing 300 mM NaCl and 200 mM imidazole (pH 8.0). The LOX activity of the elution fractions was tested employing the spectrophotometric activity assay (measuring the time dependent increase in absorbance at 235 nm). The fractions containing the catalytically active protein were pooled and frozen after addition of 10% glycerol. Typically, an electrophoretic purity of 90–95% of the enzyme preparation was reached. Specific activity of purified ALOX15 fractions normalized to 100% iron load was of the same order as described previously [43,47] for bacterial cultivation in flasks.

#### 4.2.3. Liposome Preparations

Liposomes were prepared using the classical lipid film hydration method. In order to prepare lipid bilayer, phosphatidylcholine (PC) was first dissolved in chloroform. The lipid solution was sonicated for 2 min in an ultrasound bath 200 W/50 kHz (Shenzhen DeKang Electronic, Shenzhen, China) at 38 °C. The organic solvent was then slowly removed from clear lipid solution under the flow of the argon until the lipid film is formed. Residual traces of the solvents were removed at reduced pressure (0.1 mBar) at room temperature. The lipid bilayer was hydrated with 1 mL of MilliQ water to obtain multillamellar liposomes that were further subjected to sonification for 15 min at 70 °C to yield monollamellar liposomes. Finally, the liposomal solution was subjected to a sterile filtration (0.45 µm pore size) and stored at a temperature of 4 °C prior to use. The measurement of the size and the index of polydispersity (PDI) was carried out by the laser dynamic light scraping (Delsa™ Nano, Beckman Coulter, San Jose, CA, USA) at a final concentration of 1 mM. The formed liposomes showed the average size distribution of 35.37 ± 7.3 nm and low PDI 0.200 as an indicator of a homogeneous population of phospholipid vesicles.

#### 4.2.4. Rabbit ALOX15 Kinetic Assay

For purified enzyme preparation ALOX15 activity was assayed spectrophotometrically recording the time-dependent increase in absorbance at 235 nm (Shimadzu UV-1800 photometer, Kyoto, Japan) in the substrate concentration range of 1 to 50 μM in a 1 mL quartz cuvette. The assay mixture was a PBS (pH 7.4) containing ALOX15 (2.5–5.0 μg of purified rabbit enzyme) preincubated either with a vehicle or 15 µM PC-based liposomes (final concentration). Unless specified elsewhere, the reaction was started by the addition of substrate to the incubation mixture. For this purpose, different aliquots of an aqueous sodium salt solution of the fatty acid substrate (5 mM) in PBS were applied. All measurements were carried out at room temperature as triplicates. An incubation of the enzyme with liposomes without any substrate was used as a negative kinetic control. No conjugated dien formation (increase of absorbance at 235 nm) was observed in this case.

#### 4.2.5. Inhibitor Potency Assay

The impact of inhibitors on the rate of oxygenation of linoleic acid [LA] or arachidonic acid [AA] (25 µM final concentrations) were assayed spectrophotometrically measuring the increase in absorbance at 235 nm. The assay mixture was a 0.1 M phosphate buffer, pH 7.4 containing various concentrations of inhibitors. For this purpose, compounds tested were reconstituted in DMSO and serial dilutions were carried out so that from each dilution, 1 µL was applied for the measurement. Purified rabbit ALOX15 [5 µg, specific activity with LA-25 s^−1^, electrophoretical purity (>98%)] was preincubated with a testing compound for 1 min and the reaction was started by the addition of the substrate. The linear part of the kinetic progress curve was evaluated and the activity of the solvent controls (DMSO) was set as 100%. All measurements were carried out at room temperature as triplicates.

#### 4.2.6. Molecular Docking Studies

The program GOLD5.8.0 [48] was employed to perform all docking calculations. Due to the bulkier nature of compound **2** (tricyclic pharmacophore core), no solution could be obtained for compound **2** inside the binding cavity of monomer A of the rabbit ALOX15 crystallographic structure (PDB entry 2P0M) [28]. Consequently, for this newly considered inhibitor, compound **2**, and octyl (N-(3-(1*H*-indol-2-yl)phenyl)sulfamoyl)carbamate [27], used as a bias control, the calculations were restricted to the structure of the binding cavity of monomer A of rabbit ALOX15 adapted to accommodate compound **1** [27] once this ligand was removed. Specifically, the relaxed structure corresponds to the final conformation of the 200 ns MD simulation of rabbit ALOX15 dimeric complex when compound **1** is bound to its monomer A. As could be tested before [27], this procedure is not necessary for compound **1**. Once inhibitor **2** was successfully docked inside the relaxed structure of monomer A, an MD simulation was run with the resulting complex to ensure that this structure does not provoke any bias concerning allosteric effects. Hydrogen coordinates were generated with the H++ web server [49,50] using a pH of 6.0 for titrable residues. Docking calculations for AA and LA were restricted to the binding cavity of monomer B of several relaxed rabbit ALOX15 dimeric complexes when compound **2** is bound to monomer A, that is, the structures of the MD simulation of this complex that correspond to 100 ns, 200 ns, 300 ns, and 400 ns, respectively. When compound **2** was docked, the binding site cavity used in the docking runs was a 20 Å radius sphere centered around the iron atom of monomer A. For the AA and LA docking protocol, this cavity was centered around the iron atom of monomer B. The receptor was kept fixed whereas total flexibility was given to the ligand in the conformational search. The GOLD option that considers the interactions of organic ligands with metal ions in metalloenzymes was activated, although limiting the docking exploration to hexacoordinated geometries of iron. We ensured an extensive search of the conformational space of all ligands using the most efficient genetic algorithm. The ChemScore fitness function was employed to estimate the binding free energies of ligands.

#### 4.2.7. Molecular Dynamics Simulations

The recommended procedure by the AMBER program package [51] was used to assemble all systems. The ff14SB force field was employed for the protein atoms. In contrast, the specific force field parameters of compound **2** were developed here and they were taken from previous works for AA [52], LA [53], the iron atom with its first coordination sphere [54] (His361, His366, His541, His545, Ile663 and OH^−^) and compound **1** [27]. The calculations to generate those specific parameters were carried out following the standard protocol in AMBER with Antechamber and Parmchk2 modules, even though, due to the fact that all inhibitors are far from common substrates used in AMBER, a procedure developed by MacKerell et al. [55] had to be employed with the aim of overcoming some large dihedral penalties. As the source for those parameters, the GAFF2 [51,56] library was used. The B3LYP/6-31G(d) level of theory was employed to optimize the structure of compound **2** and its atomic charges were assigned using the Merz–Kollman RESP procedure [57]. Additionally, the protonation states for all ligands were established by hand with the objective of ensuring that they match with the protonation state in physiological conditions.

All MD simulations followed the same protocol; the only differences were found in the starting structures and in the length of the production period. After combining the corresponding enzyme, inhibitor and substrate files by means of the usage of tLeap program, the different complexes obtained were solvated with an orthorhombic box of pre-equilibrated TIP3P [58] waters and their total charge was neutralized by adding sodium cations. The resulting systems contain approximately 200,000 atoms, of which around 21,000 of them belong to the protein. The remaining atoms correspond to water molecules and salt ions. All MD simulations were run with the AMBER 20 GPU (CUDA) version of the PMEMD package [59,60]. Firstly, in order to avoid close contacts, the systems were submitted to 22,000 energy minimizations steps using the steepest-descent method. In the first 6000 steps, harmonic restraints were applied to the enzyme and ligand atoms with a force constant of 5.0 kcal mol^−1^ Å^−2^, so that only the solvent and ions were relaxed exclusively. In the following 6000 steps, harmonic restraints were applied to the enzyme backbone and the substrate heavy atoms with the same force constant. In the last 10,000 steps, no restraint was applied to the whole system. After minimization, MD simulations using periodic boundary conditions were carried out. The system was gradually heated from 0 K to 300 K for a period of 200 ps. Next, an MD run of 1 ns, at constant temperature and pressure (300 K, 1 bar), was calculated to adjust the volume of the orthorhombic box so that a density of around 1 g cm^−3^ was reached. Throughout the heating and compressing, harmonic restraints were applied to the protein backbone and substrate heavy atoms with a force constant of 5.0 kcal mol^−1^ Å^−2^, whereas no restraints were applied to the rest of the system. The temperature was controlled by Langevin dynamics [61], while the pressure was adjusted by the Berendsen barostat [62]. Then, an equilibration stage of 10 ns was carried out at constant temperature (300 K) and volume. Finally, a production period was run within the same isothermal–isochoric ensemble. Along the whole MD trajectory, a time step of 2 fs was used. All bonds and bends containing hydrogen atoms were constrained by the SHAKE algorithm [63]. Nonbonding interactions were calculated with a cutoff of 9 Å. As a starting structure for the MD simulations of the rabbit ALOX15 complex when compound **2** acts as an inhibitor, we used the best docking pose of the two clusters obtained for compound **2** into monomer A of the adapted dimeric structure, described in the previous section, in which the pharmacophore core of this compound was inside the protein pocket. On the other hand, the best docking pose of octyl (N-(3-(1*H*-indol-2-yl)phenyl)sulfamoyl) carbamate bound to monomer A of the relaxed dimeric structure was employed as the starting structure for the MD simulation of the complex formed by this inhibitor and the enzyme. The length of the production period for those MD trajectories was 400 ns to relax the system in the presence of the corresponding inhibitor in each case. The structures corresponding to 100 ns, 200 ns, 300 ns and 400 ns of the MD simulation of the rabbit ALOX15 complex when compound **2** is found inside its monomer A with the inhibitor pyridine group placed at the bottom of the cavity were then taken as receptors for docking AA and LA into monomer B. Next, the best docking pose of AA and LA into monomer B for the structure corresponding to 200 ns was selected as a starting structure for the MD simulation of the complex formed by rabbit ALOX15 and the corresponding substrate. This choice was made in order to reproduce as much as possible the AA and LA binding mode observed when compound **1** acted as an inhibitor [27]. In these two cases, a production period of 200 ns was calculated. For the sake of comparison, the results from our previous studies of the MD simulations of the complexes formed by rabbit ALOX15, with compound **1** and the substrates considered are also included [27]. Analysis of the MD simulations was carried out with AmberTools18, whereas visualization of those trajectories was performed with VMD [64] and USCF CHIMERA [65] programs. The cavity surfaces were calculated with the CavityPlus web server [66].

## 5. Conclusions

The major goal of the study was to answer two questions: (i) How is inhibitor binding at the active site of the allosteric monomer translated into the catalytic monomer of the ALOX15 dimer; and (ii) How does a targeted chemical modification impact the enzyme–inhibitor interaction. For this purpose, we selected the chemical structures two previously reported pharmacophores, 5-(4-methoxyphenyl)-1*H*-indole (**1**) and 5-(4-methoxyphenyl)-1*H*-imidazole (**2**) derivatives, that exhibit substrate-selective inhibition of ALOX15. CH-to-N exchange (compounds **1** vs. **3**) reduces the affinity of the inhibitor to the enzyme, but retains its substrate selective character against linoleic acid oxygenation. In turn, 5-(4-methoxyphenyl)-1*H*-imidazole derivative **2** induced weaker drug responses to linoleate oxygenase activity of ALOX15. The results of our docking experiments and the molecular dynamics simulations indicated that not only the presence of the MeO-group, but also its position inside the binding pocket of monomer A appears to be important for allosteric inhibition of ALOX15. Our experimental data correlate with the theoretical results predicted by computational modelling and reveal novel aspects in the structure–activity relationship among lipoxygenase inhibitors.

## Figures and Tables

**Table 1:** Inhibitory activity of compounds **1**–**3** against rabbit ALOX15.

Inhibitor	IC_50_(LA), µM	IC_50_(AA), µM	IC_50_(LA)/IC_50_(AA) Ratio
**1**	0.03 ± 0.02	2.79 ± 0.18	0.010
**1** + PC	0.06 ± 0.04	3.45 ± 0.10	0.018
**2**	1.8 ± 1.2	54.02 ± 2.50	0.032
**3**	0.16 ± 0.02	13.21 ± 1.59	0.011

**Table 2:** Main interactions of the representative structure of the most populated cluster * for compound **2**.

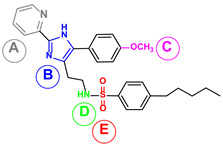			
Group	Type of Interaction	Interaction Partner	Distance
A	π-π	His361 (sidechain) His366 (sidechain)	
B, NH	hydrogen bond	Fe(III)-OH^−^	d(H1-OH) = 1.982 Å
C, CH_3_O	hydrogen bonds	Arg403 (sidechain NH_2_) Gln596 (sidechain NH_2_) Gln601 (sidechain NH_2_ and backbone NH)	d(O1-HH12-Arg403) = 3.793 Å d(O1-HH22-Arg403) = 4.301 Å d(O1-HE21-Gln596) = 5.451 Å d(O1-HE22-Gln596) = 5.229 Å d(O1-HE22-Gln601) = 4.341 Å d(O1-H-Gln601) = 5.237 Å
D, NH	hydrogen bond	Arg403	d(N4-HH11-Arg403) = 3.254 Å
E, SO_2_	hydrogen bond	Leu408 (backbone NH) Gln596 (sidechain NH_2_)	d(O3-H-Leu408) = 3.421 Å d(O2-HE21-Gln596) = 3.731 Å

* Cluster analysis has been performed to determine the most characteristic features of the binding mode of compound 2 with allosteric effects. The cluster analysis has been performed over its MD simulation using an RMSD of 0.5 Å for the backbone atoms of the inhibitor. Three different clusters were obtained, one of them significantly more populated (84.4%).

**Table 3:** Main interactions of the representative structure of the most populated cluster * for compound **1**.

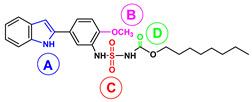			
Group	Type of Interaction	Interaction Partner	Distance
A, NH	hydrogen bond	Fe(III)-OH^−^	d(H1-OH) = 2.116 Å
B, CH_3_O	hydrogen bonds	Gln596 (sidechain NH_2_) Arg403 (sidechain NH_2_)	d(O1-HE22-Gln596) = 3.534 Å d(O1-HH11-Arg403) = 4.465 Å
B, CH_3_O	electrostatic	Arg599	**
C, SO_2_	hydrogen bond	Gln596 (side chain NH_2_)	d(O2-HE21-Gln596) = 2.059 Å
D, CO	hydrogen bond	Leu408 (backbone NH)	(O4-H-Leu408) = 2.345 Å

* Cluster analysis has been performed to determine the most characteristic features of the binding mode of compound 1 with allosteric effects. The cluster analysis has been performed over its MD simulation using an RMSD of 0.5 Å for the backbone atoms of the inhibitor. Four different clusters were obtained, one significantly more populated (60.0%). ** To a greater or lesser extent, the whole Arg599 sidechain interacts electrostatically with the MeO-group of compound **1**, so that several distances can be employed to characterise this interaction.

**Table 4:** Percentage of well-oriented structures for H_13_- and H_11_- hydrogen abstractions from AA and LA, respectively, with compounds **1** or **2** bound to monomer A. A well-oriented structure is that which has at least one hydrogen atom well-oriented for the hydrogen abstraction, in other words, the hydrogen atom to be abstracted is closer to the Fe(III)-OH^−^ cofactor than the carbon atom that is attached to it.

Substrate	No Inhibitor	Compound 2	Compound 1
AA	97.78	93.38	87.90
LA	90.09	84.80	34.00

## Data Availability

The data presented in this study is contained within the article.

## Supplementary Materials

The following supporting information can be downloaded at: https://www.mdpi.com/article/10.3390/molecules28145418/s1: docking studies and MD simulations of enzyme–inhibitor complexes (Figure S1: MD simulations of dimeric rabbit ALOX15 complex; Figure S2: Environment of Arg403 and Arg599 of monomer B in the presence of inhibitors in monomer A; Figure S3: Cavity surfaces of monomer B when compound **1** (A) and compound **2** (B) are bound to ALOX15 conformer A as well as spectral and analytical data (^1^H and ^13^C NMR spectra, MS spectra and HPLC data).

## Sample Availability

Samples of the compounds **1**–**3** are available from the authors.
