# Supplementary material for: Different Structures—Similar Effect: Do Substituted 5-(4-Methoxyphenyl)-1H-indoles and 5-(4-Methoxyphenyl)-1H-imidazoles Represent a Common Pharmacophore for Substrate Selective Inhibition of Linoleate Oxygenase Activity of ALOX15?
Source: Molecules. 2023 Jul 14;28(14):5418. doi: 10.3390/molecules28145418 (PMC10383952; doi:10.3390/molecules28145418)
Supplement: Supplementary file 1 [file molecules-28-05418-s001.zip › molecules-2435725-supplementary.pdf]

molecules

MDPI

Supporting Information

Different structures – similar effect. Do substituted 5-(4-methoxyphenyl)-1*H*-indoles and 5-(4-methoxyphenyl)-1*H*-imidazoles represent a common pharmacophore for substrate selective inhibition of linoleate oxygenase activity of ALOX15?

Alexander Zhuravlev [1], Alejandro Cruz [2], Vladislav Aksenov [1,3,] Alexey Golovanov[1], José M. Lluch [2,4], Hartmut Kuhn [5], Àngels González-Lafont [2,4] and Igor Ivanov [1*]

1 Lomonosov Institute of Fine Chemical Technologies, MIREA - Russian Technological University, Vernadskogo pr. 86, 119571 Moscow, Russia; igor_ivanov@gmx.de (I.I.); alekszhur95@yandex.ru (A.Z.), aksenov.v.v@edu.mirea.ru (V.A)

2 Departament de Química, Universitat Autònoma de Barcelona, 08193 Bellaterra, Barcelona, Spain; Alejandro.Cruz@uab.cat (A.C.)

3 Shemyakin-Ovchinnikov Institute of Bioorganic Chemistry, Miklihio-Maklaja str, 16/10c4, 117997, Moscow, Russia

4 Institut de Biotecnologia i de Biomedicina (IBB), Universitat Autònoma de Barcelona, 08193 Bellaterra, Barcelona, Spain; JoseMaria.Lluch@uab.cat (J.M.L.); Angels.Gonzalez@uab.cat (A.G.-L.)

5 Institute of Biochemistry, Charite - University Medicine Berlin, Corporate member of Free University Berlin, Humboldt University Berlin and Berlin Institute of Health, Charitéplatz 1, D-10117 Berlin, Germany; hartmut.kuehn@charite.de

* Correspondence: igor_ivanov@gmx.de; Tel.: (+7)-495-246-05-55 (supl. 884)

Table of contents

1. Supporting information to the main body text 3
2. Synthesis of N-(2-(5-(4-methoxyphenyl)-2-(pyridin-2-yl)-1H-imidazol-4-yl)ethyl)-4-pentylbenzenesulfonamide (**2**) 6
3. Spectral and analytical data 10
   3.1. ^{1}H- and ^{13}C-NMR Spectra 10
   3.2. MS spectral analysis 22

1. Supporting information to the main body text

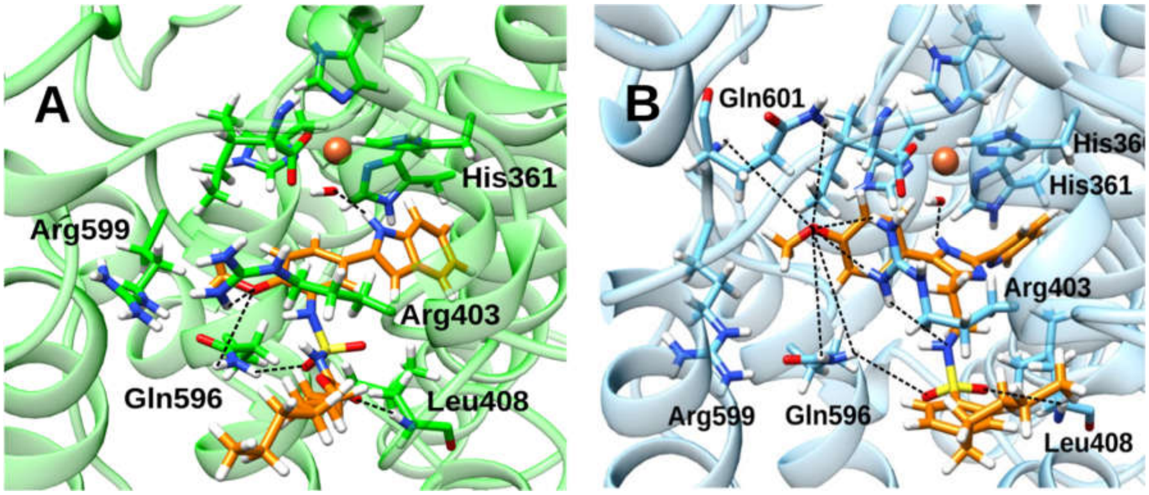

Figure S1: MD simulations of dimeric rabbit ALOX15 complex in the presence of inhibitors **1** and **2**. The binding modes of compound **1** (A) and compound **2** (B) inside the binding pocket of monomer A, when the methoxyphenyl group is located at the cavity side defined by helices $\alpha 2$ and $\alpha 18$, are depicted. Those binding modes have been determined from a clustering analysis using an RMSD of 0.5 Å for the ligand's backbone atoms.

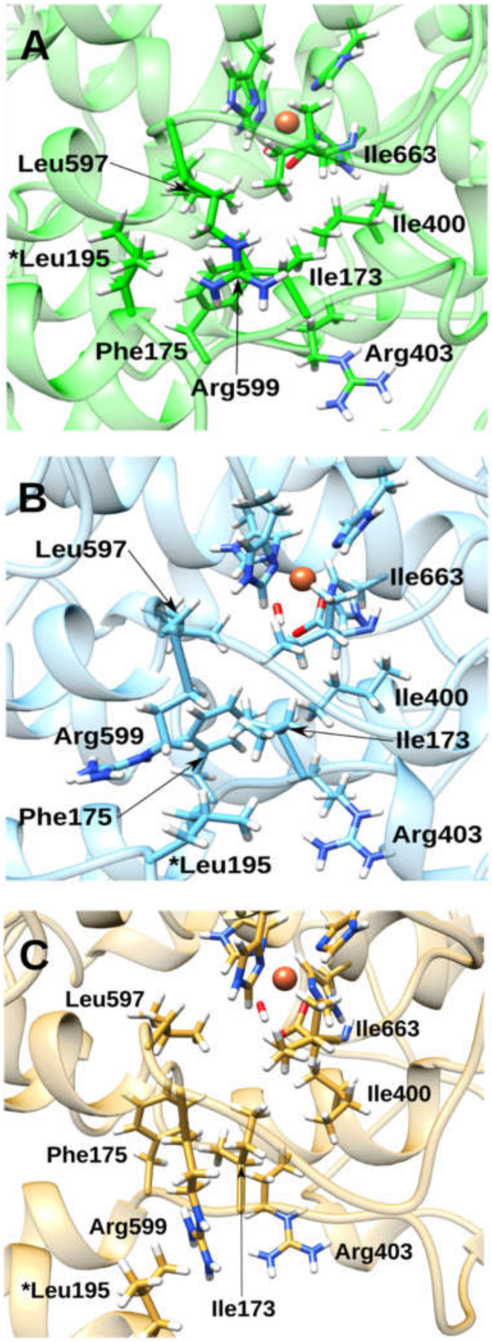

Figure S2: Environment of Arg403 and Arg599 of monomer B in the presence of compound **1** (A), compound **2** (B), and octyl (N-(3-(1*H*-indol-2-yl)phenyl)sulfamoyl)carbamate (C) in monomer A (not shown in the **Figure** S**1**). These pictures correspond to the last structure of the corresponding inhibitor-enzyme MD simulation. Although the binding mode of compounds **1** and **2,** which exhibit allosteric effects, seem similar, the interactions between the compounds and the enzyme significantly differ. That is reflected in the different blockage of Arg403 and Arg599 in (A) versus (B). For the sake of comparison, the environment of Arg403 and Arg599 is shown in (C) when an inhibitor without allosteric properties (octyl (N-(3-(1*H*-indol-2-yl)phenyl)sulfamoyl)carbamate) is bound to monomer A.

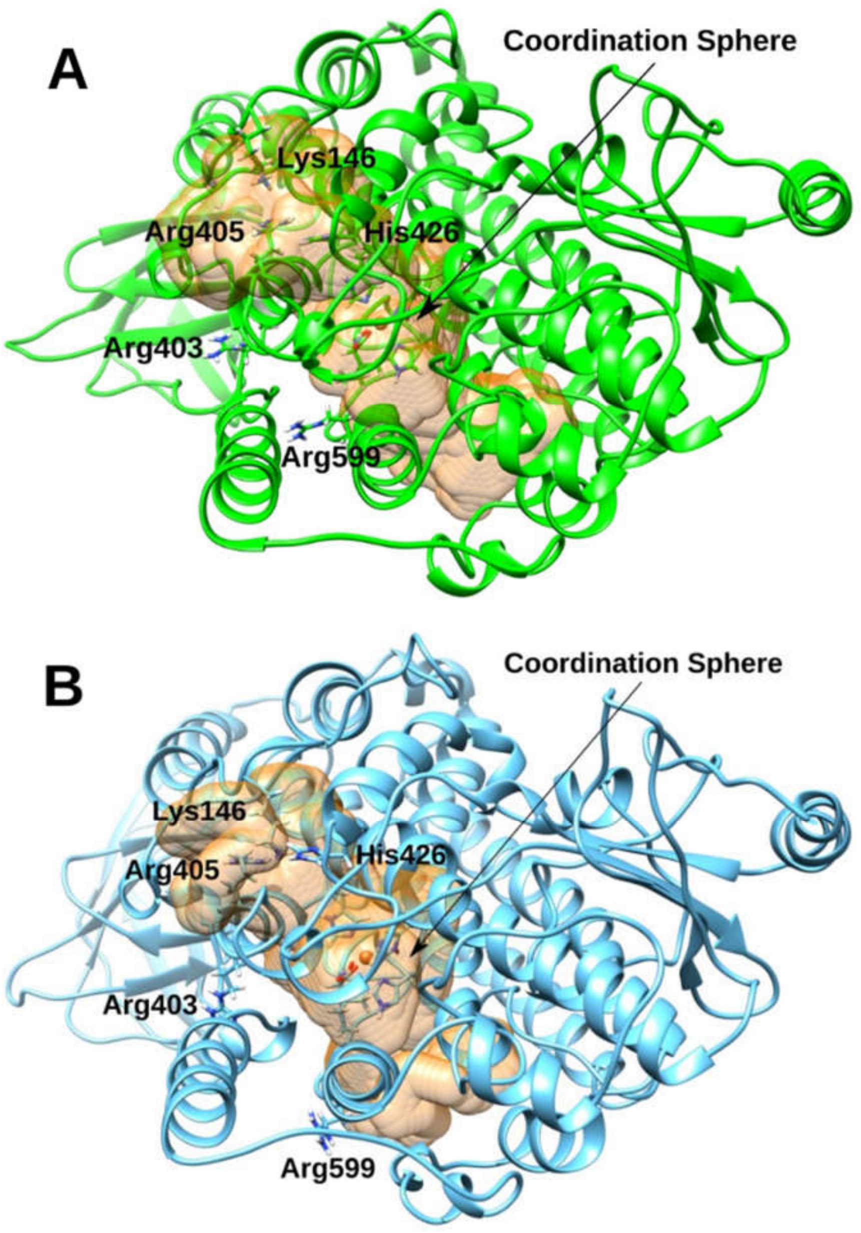

Figure S3: Cavity surfaces of monomer B when compound **1** (A) and compound **2** (B) are bound to conformer A. The cavity plotted has the best score and includes the entrance to the binding pocket and the iron coordination sphere, whereas the sidechains of Arg403 and Arg599 are pointing outward the cavity. Calculations of these cavity surfaces were carried out with the CavityPlus web server. Compound **1** in the binding pocket of monomer A modifies the shape of the substrate binding cavity of monomer B. The two Arg residues (Arg403, Arg599) that anchor the carboxylic group of the fatty acid substrate in the absence of inhibitor are blocked in this complex. The MD simulations of the dimer with compound **2** bound to monomer A show a substrate binding cavity of conformer B that is like that induced by compound **1**.

2. Synthesis of N-(2-(5-(4-methoxyphenyl)-2-(pyridin-2-yl)-1*H*-imidazol-4-yl)ethyl)-4-pentylbenzenesulfonamide (2)

Scheme S1

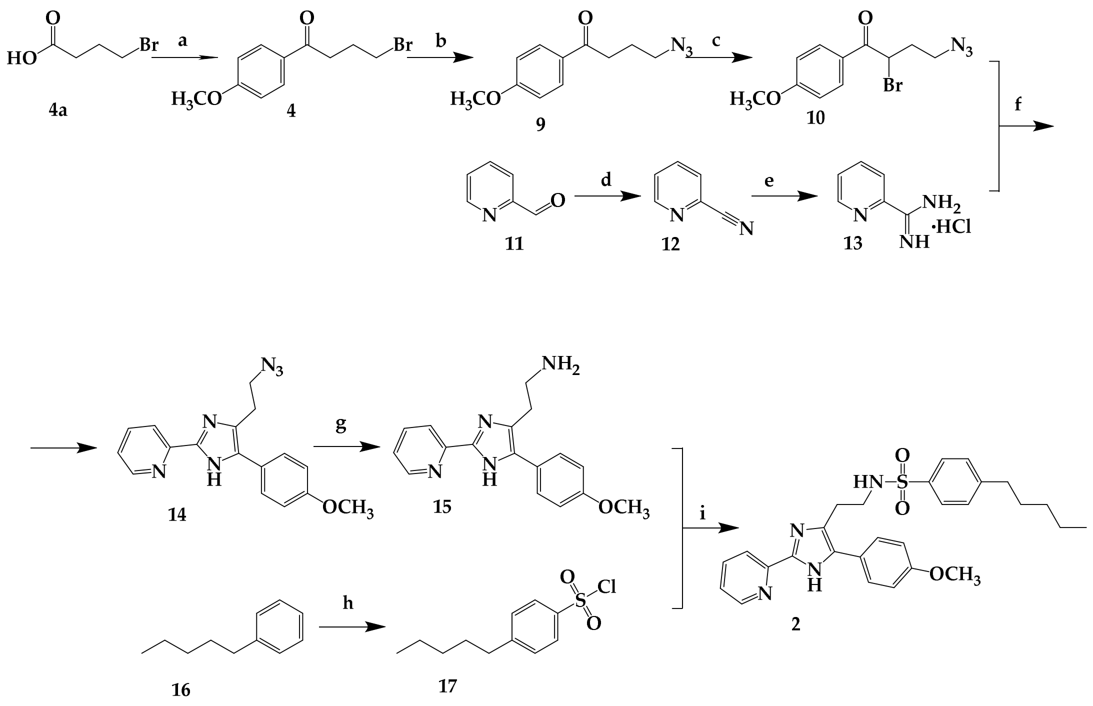

Reagents and conditions: (**a**) $(COCl)_2$, DMF, CH_2Cl_2, 0°C, 1 h; then anisole, $AlCl_3$, CH_2Cl_2, 20°C, 2 h; (**b**) NaN3, DMF, 90°C, 2,5 h, darkness; (**c**) Br_2, AcOH, r.t., 2 h, darkness; (**d**) I_2, NH_4OH, THF, r.t., 15 min; (**e**) MeONa, MeOH, r.t., overnight; then NH_4Cl, reflux, 6 h; (**f**) K_2CO_3, DMF, 90°C, 20 h; (**g**) H_2, 10% Pd/C, MeOH, r.t., 1 h; (**h**) HSO_3Cl, $CHCl_3$, 40°C, 30 min; (**i**) Et_3N, CH_2Cl_2, 1 h., rt.

4-Bromo-1-(4-methoxyphenyl)butan-1-one (4). A solution of oxalyl chloride (1.542 ml; 17.964 mmol) in 3 ml of CH_2Cl_2 and 3 drops of DMF was added dropwise to a solution of 4-bromobutanoic acid **4a** (1 g; 5.998 mmol) in 2.5 ml of CH_2Cl_2 at 0°C. After the mixture was stirred at room temperature for 1 h the volatile organic compounds were evaporated. The raw anhydride was dissolved in 4 ml of CH_2Cl_2 was then added to a solution of anisole (0.651 ml; 5.998 mmol) and $AlCl_3$ (0.876 g; 6.587 mmol) in 2 ml of CH_2Cl_2 at 0°C. The resulting mixture was stirred for 3h at 20°C and then quenched with H_2O (20 ml). Organic products were extracted with CH_2Cl_2 (3x25 ml). Combined organic extracts were dried over Na_2SO_4 and concentrated in vacuum. The raw product was purified by column chromatography on silica gel using an isocratic eluent system (Pet/EtOAc, 3:1 by vol.). Yield: 86.7 %. R_f = 0.58 (Pet/EtOAc, 3:1 by vol.). ^{1}H NMR (600 MHz, $CDCl_3$) δ = 7.96 (dt, J = 9.0 Hz, 2H), 6.94 (dt, J = 9.0 Hz, 2H), 3.87 (s, 3H), 3.54 (t, J = 6.4 Hz,

2H), 3.12 (t, J = 6.9 Hz, 2H), 2.29 (p, J = 6.6 Hz, 2H). ^{13}C NMR (75 MHz, $CDCl_3$) δ = 27.23, 33.89, 36.32, 55.62, 113.92, 130.02, 130.43, 163.73, 197.50.

4-Azido-1-(4-methoxyphenyl)butan-1-one (9). Sodium azide (284 mg; 4.361 mmol) was added to a solution of 4-bromo-1-(4-methoxyphenyl)butan-1-one (**4**) (747 mg; 2.907 mmol) in 5 ml DMF and the mixture was stirred for 2.5 h at 90°C in total darkness. The mixture was quenched with 13 ml saturated NH_4Cl solution, organic products were extracted with CH_2Cl_2 (3x20 ml). The combined organic extracts were dried over Na_2SO_4 and concentrated in vacuum. The raw product was purified by column chromatography on silica gel using an isocratic eluent system (Pet/EtOAc, 3:1 by vol.). Yield: 85,4%. R_f = 0.43 (Pet/EtOAc, 3:1 by vol.). ^1H NMR (300 MHz, $CDCl_3$) δ = 7.98 – 7.92 (m, 2H), 6.99 – 6.88 (m, 2H), 3.87 (s, 3H), 3.41 (t, J = 6.6 Hz, 2H), 3.04 (t, J = 7.1 Hz, 2H), 2.03 (p, J = 6.8 Hz, 2H). ^{13}C NMR (75 MHz, $CDCl_3$) δ 23.58, 34.84, 51.02, 55.53, 113.85, 129.92, 130.32, 163.65, 197.54.

4-Azido-2-bromo-1-(4-methoxyphenyl)butan-1-one (10). To a solution of 4-azido-1-(4-methoxyphenyl)butan-1-one (**9**) (274 mg; 1.251 mmol) in 6.3 mL of AcOH Br_2 (200 mg, 1.251 mmol) was added dropwise and the mixture was stirred at room temperature for 2 hours until the reaction mixture became colorless. The volatile compounds were evaporated in vacuum, EtOAc added (20 ml), and the organic layer was washed with 1 M HCl (3x25 ml), sat. solution of NH_4Cl (3x25 ml), brine (3x25 ml), dried over Na_2SO_4 and concentrated in vacuum. The raw product was purified by column chromatography on silica gel in an isocratic eluent system (Pet/EtOAc, 95:5 by vol.). Yield: 66.0%. R_f = 0.60 (Pet/EtOAc, 3:1 by vol.). ^1H NMR (300 MHz, $CDCl_3$) δ = 8.00 (d, J = 9.1 Hz, 2H), 6.96 (d, J = 9.0 Hz, 2H), 5.31 – 5.24 (m, 1H), 3.88 (s, 3H), 3.63 – 3.52 (m, 2H), 2.40 – 2.31 (m, 2H). ^{13}C NMR (75 MHz, $CDCl_3$) δ = 32.76, 43.78, 49.16, 55.68, 114.16, 126.82, 131.46, 164.25, 191.16.

2-Cyanopyridine (12). Iodine crystals (261 mg; 1.030 mmol) were added to a solution of 2-pyridinecarboxyaldehyde (**11**) (100 mg; 0.9336 mmol) in 8 ml of NH_4OH and 1 ml of THF and the mixture was kept for 15 min at room temperature until the reaction mixture became colorless. The reaction mixture was quenched with 5% $Na_2S_2O_3$ (5 ml) and the products were extracted with Et_2O (3x20 ml). Combined organic extracts were dried over Na_2SO_4 and concentrated in vacuum. The raw product was used on the next step without further purification. Yield: 90.7%. MS (EI, 70eV, m/z): 104.10 (100%), 77.08 (56%), 50.08 (25%).

Picolinimidamide hydrochloride (13). A mixture of sodium methoxide (5 mg; 0.0846 mmol) and 2-cyanopyridine (**12**) (88 mg; 0.846 mmol) in 0.88 ml MeOH was stirred overnight at room temperature under the atmosphere of argon. After

NH_4Cl (5 mg; 0.0846 mmol) was added to the reaction the mixture was refluxed for 6 hours. The product was sedimented by adding 100 μl Et_2O to give white crystals. Yield 95.0%. ^1H NMR (300 MHz, DMSO-d_6) δ = 9.54 (s, 4H), 8.82 (ddd, J = 4.7, 1.8, 0.9 Hz, 1H), 8.42 (d, J = 8.0 Hz, 1H), 8.16 (td, J = 7.9, 1.7 Hz, 1H), 7.79 (ddd, J = 7.6, 4.7, 1.0 Hz, 1H). ^{13}C NMR (75 MHz, DMSO-d_6) δ = 123.48, 128.52, 138.28, 143.92, 149.86, 162.20.

2-(4-(2-azidoetyl)-5-(4-methoxyphenyl)-1*H*-imidazol-2-yl)pyridine (14). K_2CO_3 (142 mg; 1.025 mmol) was added to a precooled mixture of 4-azido-2-bromo-1-(4-methoxyphenyl)butan-1-one (**10**) (246 mg; 0.825 mmol) and picolinimidamide hydrochloride (**13**) (108 mg; 0.684 mmol) in 2.6 ml DMF and the mixture was stirred for 20 h at 90°C. The reaction was quenched with 5 ml H_2O, the organic products were extracted with EtOAc (3x30 ml). The combined organic extracts were washed with H_2O (2x25 ml), saturated solution of $NaHCO_3$ (3x25 ml), brine (3x25 ml), dried over Na_2SO_4 and concentrated in vacuum. The raw product was purified by silica gel column chromatography using an isocratic eluent system (Pet/EtOAc/NH_4OH, 50:25:1 by vol.). Yield 57.6%. R_f = 0.36 (Pet/EtOAc/NH_4OH, 50:25:1 by vol.). ^1H NMR (300 MHz, acetone-d_6) δ = 8.53 (ddd, J = 4.9, 1.8, 1.0 Hz, 1H), 8.16 (dt, J = 8.0, 1.1 Hz, 1H), 7.84 (ddd, J = 8.0, 7.5, 1.7 Hz, 1H), 7.67 (d, J = 8.7 Hz, 2H), 7.29 (ddd, J = 7.5, 4.9, 1.2 Hz, 1H), 7.05 – 6.96 (m, 2H), 3.83 (s, 3H), 3.73 (t, J = 7.1 Hz, 2H), 3.14 (t, J = 7.3 Hz, 2H). ^{13}C NMR (75 MHz, acetone-d_6) δ = 51.43, 55.56, 114.76, 120.29, 123.59, 129.33, 137.69, 145.63, 149.83, 149.99, 159.78.

2-(5-(4-methoxyphenyl)-2-(pyridin-2-yl)-1*H*-imidazol-4-yl)ethan-1-amine (15). 10% Pd/C (10 mg, 10% by mass) was added to a solution of 2-(4-(2-azidoetyl))-5-(4-methoxyphenyl)-1*H*-imidazol-2-yl)pyridine (**14**) (96 mg; 0.3 mmol) in 10 ml of MeOH and the mixture was stirred for 1 h at room temperature in the atmosphere of H_2. The catalyst was filtered off, washed with MeOH (3x15 ml) and the combined metanolic solution was concentrated in vacuum. The raw product was purified by column chromatography on silica gel in an isocratic eluent system (MeOH/NH_4OH, 20:1 by vol.). Yield 75.0%. R_f = 0.33 (MeOH/NH_4OH, 20:1). ^1H NMR (300 MHz, CD_3OD) δ = 8.56 (ddd, J = 4.9, 1.8, 1.0 Hz, 1H), 8.06 (dt, J = 8.0, 1.1 Hz, 1H), 7.82 (td, J = 7.8, 1.8 Hz, 1H), 7.55 – 7.44 (m, 2H), 7.28 (ddd, J = 7.5, 4.9, 1.2 Hz, 1H), 7.03 – 6.92 (m, 2H), 3.80 (s, 3H), 3.05 – 2.85 (m, 4H). ^{13}C NMR (75 MHz, DMSO-d_6) δ = 28.40, 41.00, 55.05, 113.79, 119.27, 122.61, 127.95, 137.03, 143.76, 148.80, 148.86, 157.88.

4-Pentylbenzenesulfochloride (17). A solution of chlorosulfonic acid (224 μl, 3.378 mmol) in 3.5 ml $CHCl_3$ was added dropwise to a precooled to 0°C solution

of pentylbenzene (**16**) (100 mg; 0.676 mmol) in 3.5 ml of $CHCl_3$ and the reaction mixture was stirrred for 30 min at 40°C. The reaction was quenched with with 25 ml brine. Organic products were extracted with $CHCl_3$ (3x25 ml), dred over Na_2SO_4 and concentrated in vacuum. The product was used without further purification. Yield 85.5%. R_f = 0.18 (Pet/EtOAc, 50:1 by vol.). ^{1}H NMR (300 MHz, $CDCl_3$) δ = 7.94 (dm, J = 8.6 Hz, 2H), 7.41 (dm, J = 8.7 Hz, 2H), 2.72 (t, J = 7.6 Hz, 2H), 1.66 (p, J = 7.6 Hz, 2H), 1.38 – 1.30 (m, 4H), 0.90 (t, J = 6.9 Hz, 3H). ^{13}C NMR (75 MHz, $CDCl_3$) δ = 14.05, 22.54, 30.72, 31.45, 36.13, 127.20, 129.72, 141.90, 151.82.

N-(2-(5-(4-methoxyphenyl)-2-(pyridin-2-yl)-1*H*-imidazol-4-yl)ethyl)-4-pentylbenzosulfonamide (2). To a precooled solution of 2-(5-(4-methoxyphenyl)-2-(pyridin-2-yl)-1*H*-imidazol-4-yl)ethan-1-amine (**15**) (35 mg; 0.119 mmol) in 200 μl CH_2Cl_2 with Et_3N (26 μl; 0.179 mmol) a solution of 4-pentylbenzenesulfochloride (**17**) (36 mg; 0.143 mmol) in 450 μl of CH_2Cl_2 was added dropwise and the mixture was stirred for 1 h at room temperature. The reaction was quenched with 1M HCl (3x20 ml). Organic products were extracted with CH_2Cl_2 (3x25 ml). The combined organic extracts were washed with saturated solution of $NaHCO_3$ (3x25 ml), brine (3x25 ml), dried over Na_2SO_4 and concentrated in vacuum. The raw product was purified by silica gel column chromatography using isocratic eluent system (EtOAc/Pet/NH_4OH, 400:50:9 by vol). Yield 88.3%. R_f = 0.55 (EtOAc/Pet/NH_4OH, 400:50:9 by vol.). Analytical HPLC: Rt = 8.3 min (235 nm), Agilent PoroShell300-SB C18 (75x2.1, 7μm) using the following gradient using mobile phase A (water containing 10mM ammonium formate,with 0.1% formic acid) and mobile phase B (acetonitrile) at a flow rate of 0.5 mL/min. The elution scheme was as follows: isocratic at 10% of phase B, linear increase of mobile phase B from 10 to 98 % in 8 min, isocratic at 98% of phase B for 2 min and linear decrease of mobile phase B from 98 to 10 % in 4 min. ^{1}H NMR (300 MHz, $CDCl_3$) δ = 10.74 (s, 1H), 8.33 (d, J = 4.0 Hz, 1H), 8.02 (d, J = 8.0 Hz, 1H), 7.75 (d, J = 8.3 Hz, 2H), 7.68 (td, J = 8.1, 1.2 Hz, 1H), 7.27 (d, J = 8.1 Hz, 2H), 7.21 (d, J = 8.3 Hz, 2H), 7.12 (dd, J = 7.4, 4.9 Hz, 1H), 6.85 (d, J = 8.3 Hz, 2H), 6.75 (s, 1H), 3.79 (s, 3H), 3.38 – 3.29 (m, 2H), 2.87 (t, J = 6.0 Hz, 2H), 2.59 (t, J = 7.6 Hz, 2H), 1.57 (p, J = 7.3 Hz, 2H), 1.34 – 1.24 (m, 4H), 0.86 (t, J = 6.8 Hz, 3H). ^{13}C NMR (75 MHz, $CDCl_3$) δ = 14.05, 22.51, 26.63, 30.79, 31.45, 35.84, 42.89, 55.38, 114.33, 119.92, 123.01, 127.14, 128.39, 128.99, 137.14, 137.49, 144.44, 148.04, 148.71, 159.11. MS (ESI): $[M + H]^+$ = 505.30.

3. Spectral and analytical data

3.1. ¹H- and ¹³C-NMR Spectra

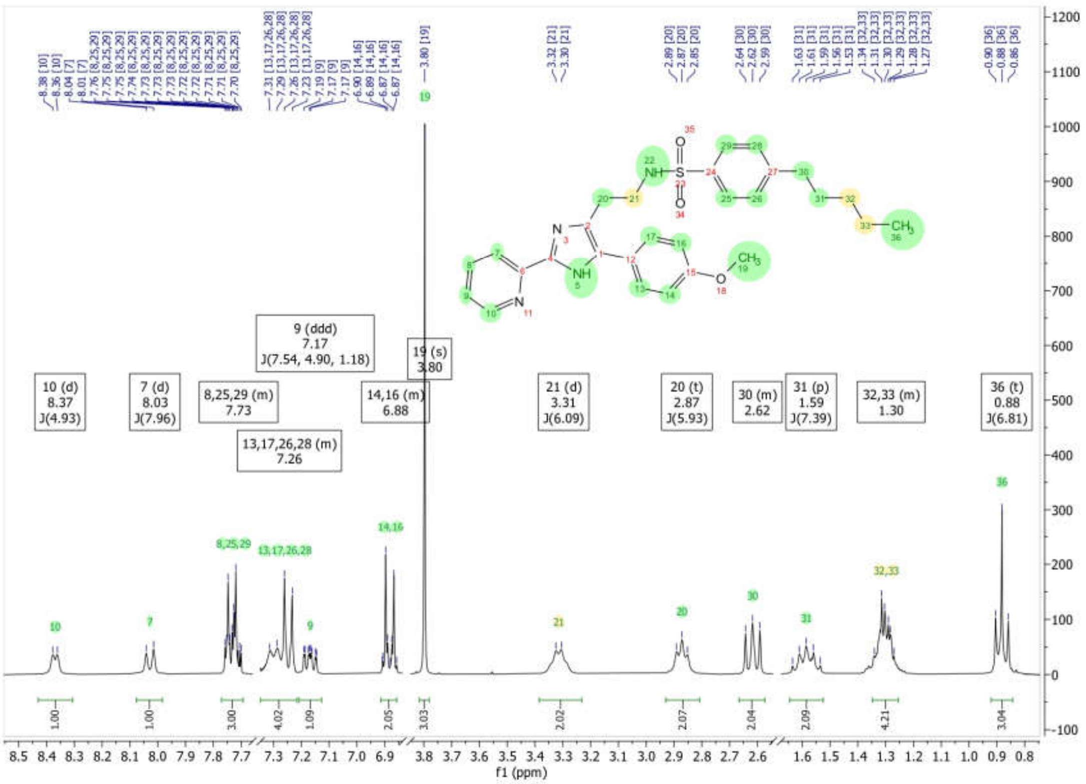

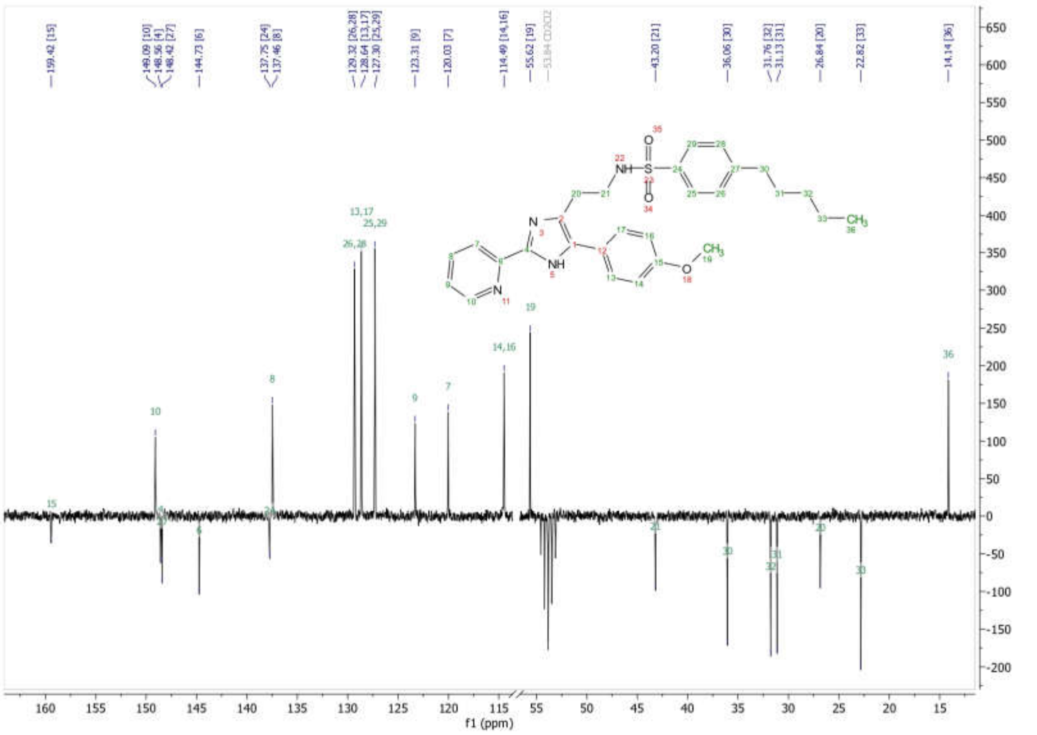

¹H- and ¹³C-NMR spectra of compound **2**

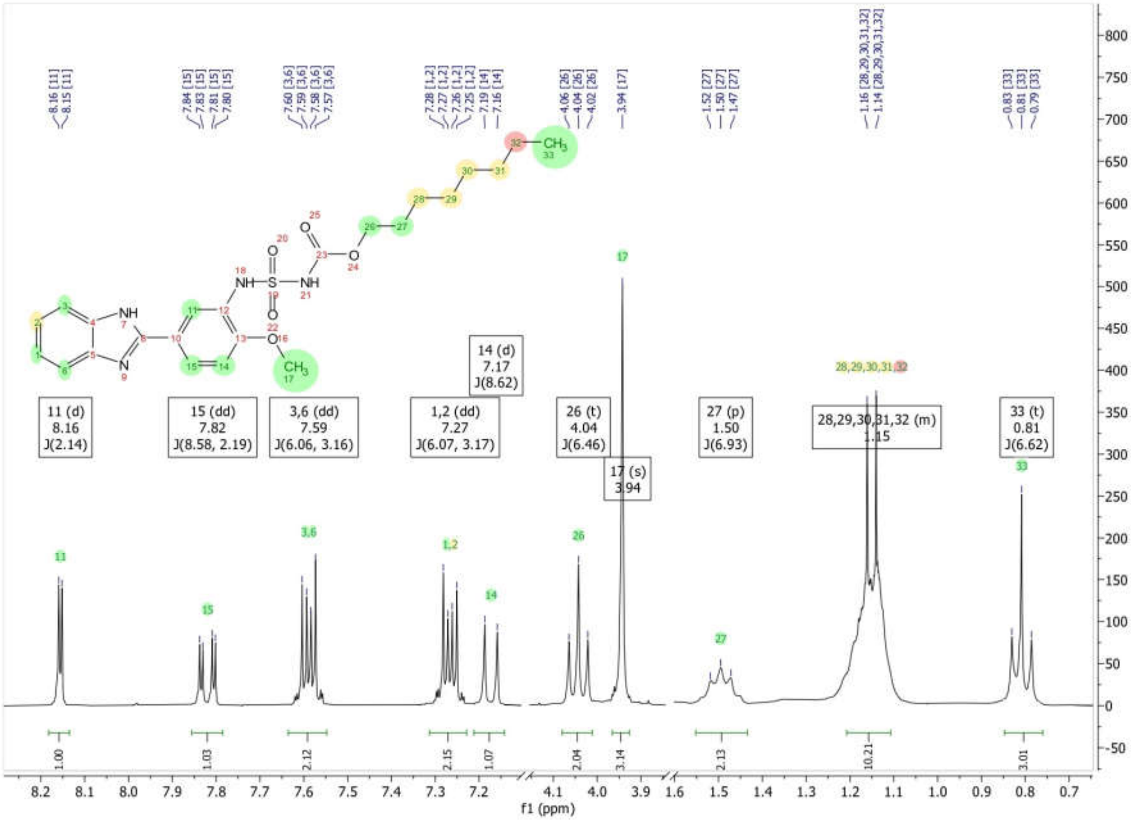

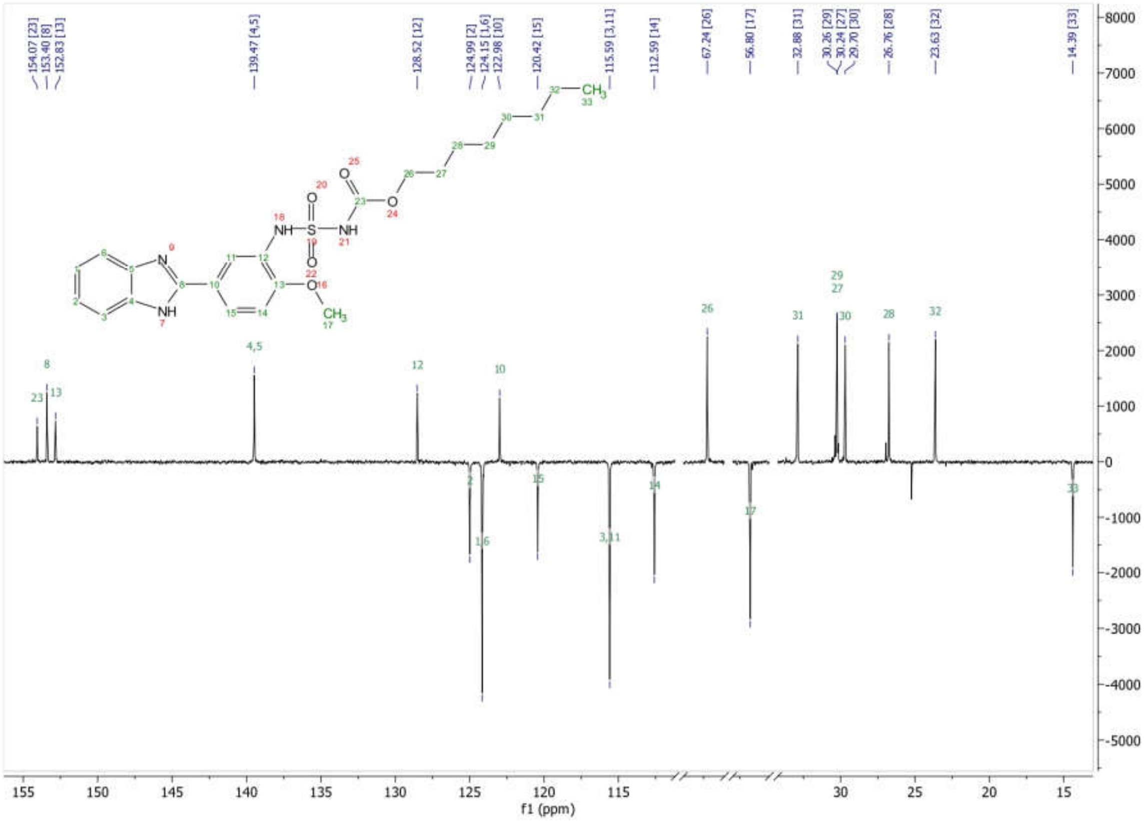

^{1}H- and ^{13}C-NMR spectra of compound **3**

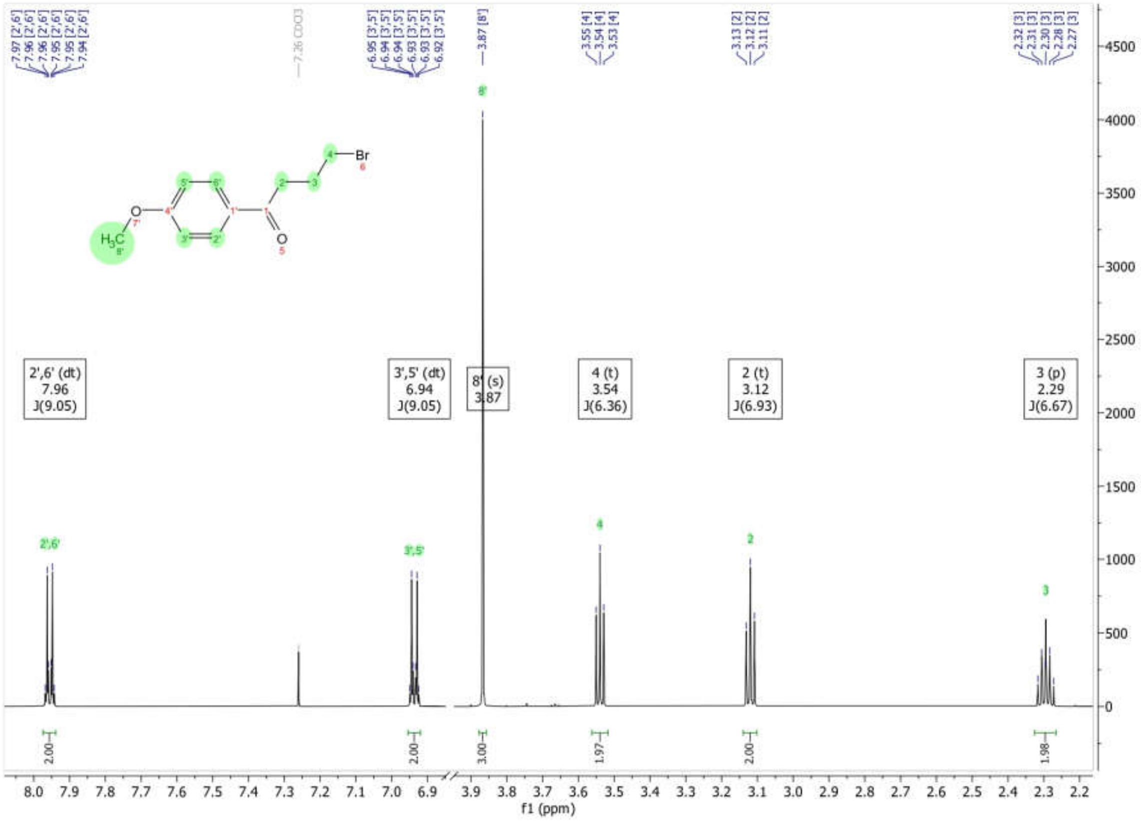

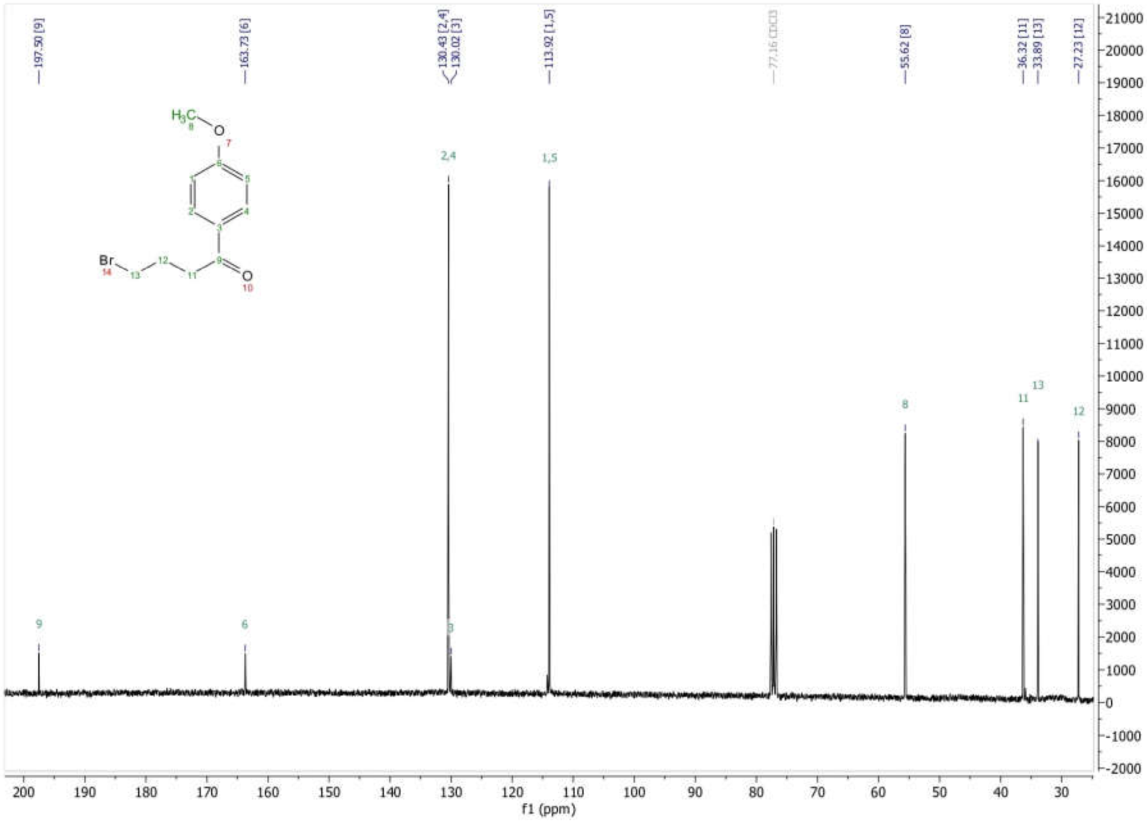

^{1}H- and ^{13}C-NMR spectra of compound **4**

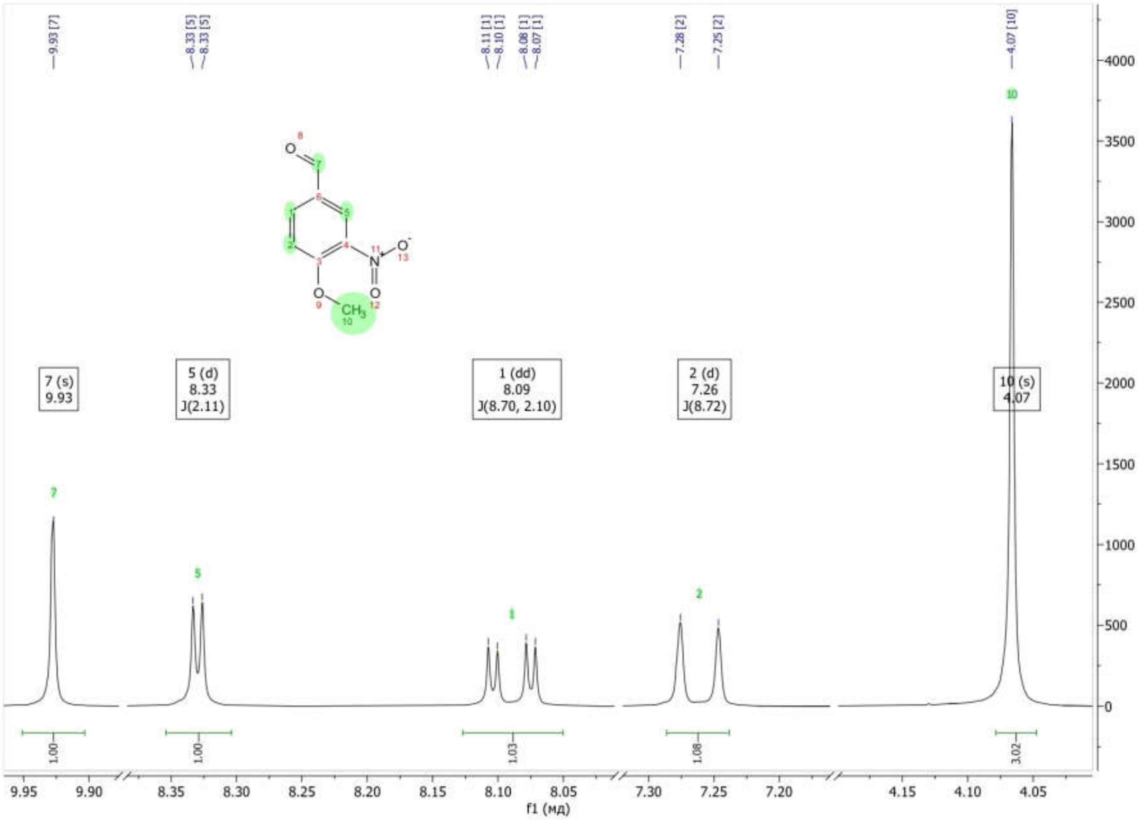

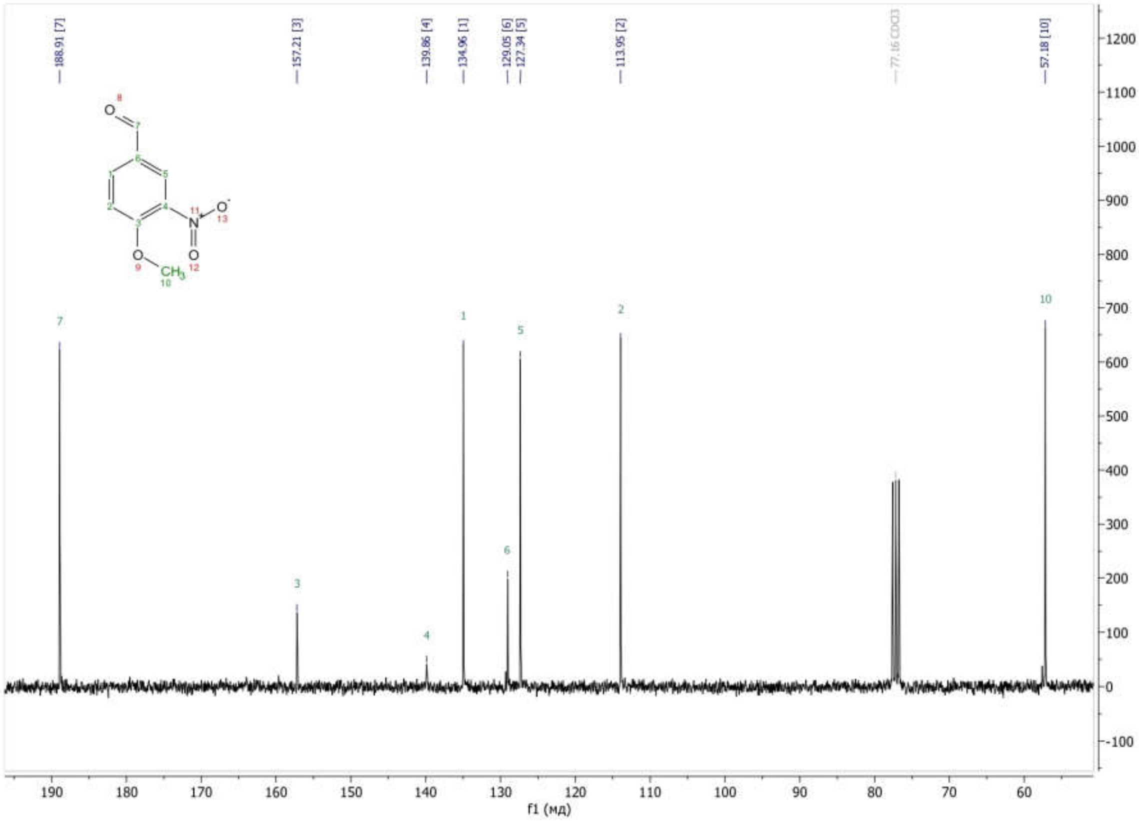

^{1}H- and ^{13}C-NMR spectra of compound **6**

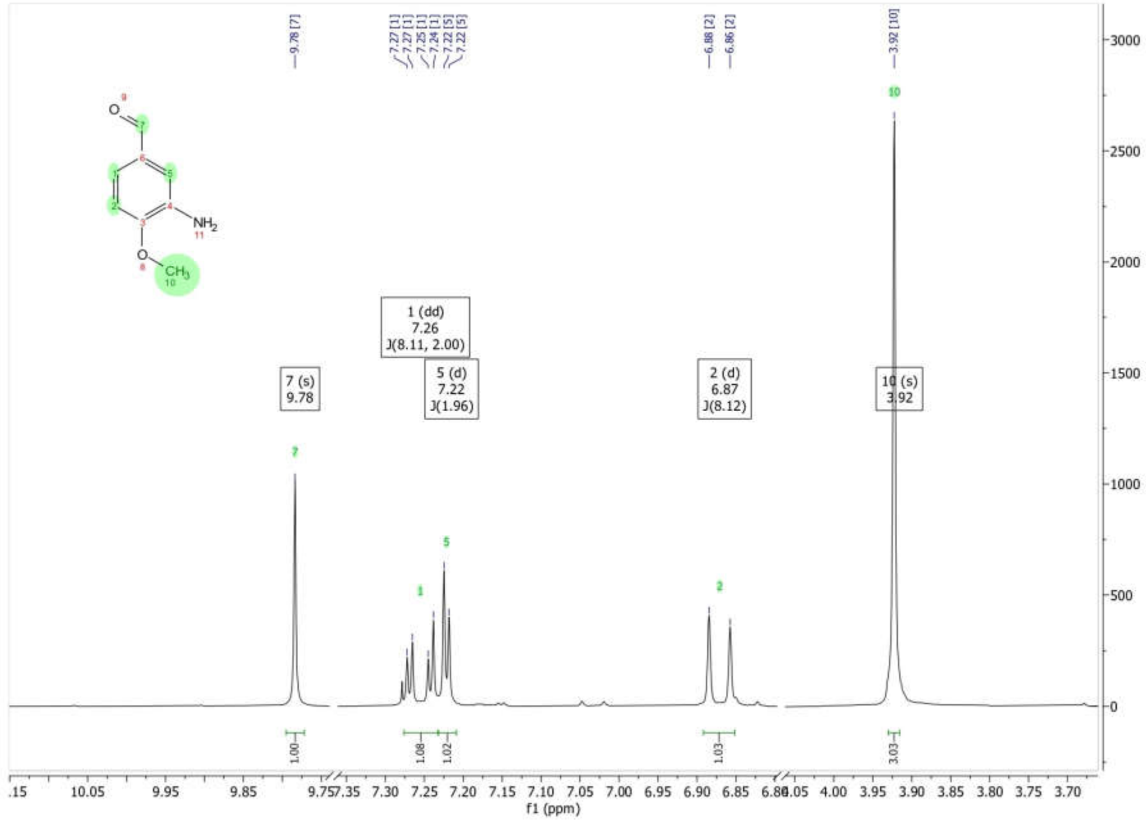

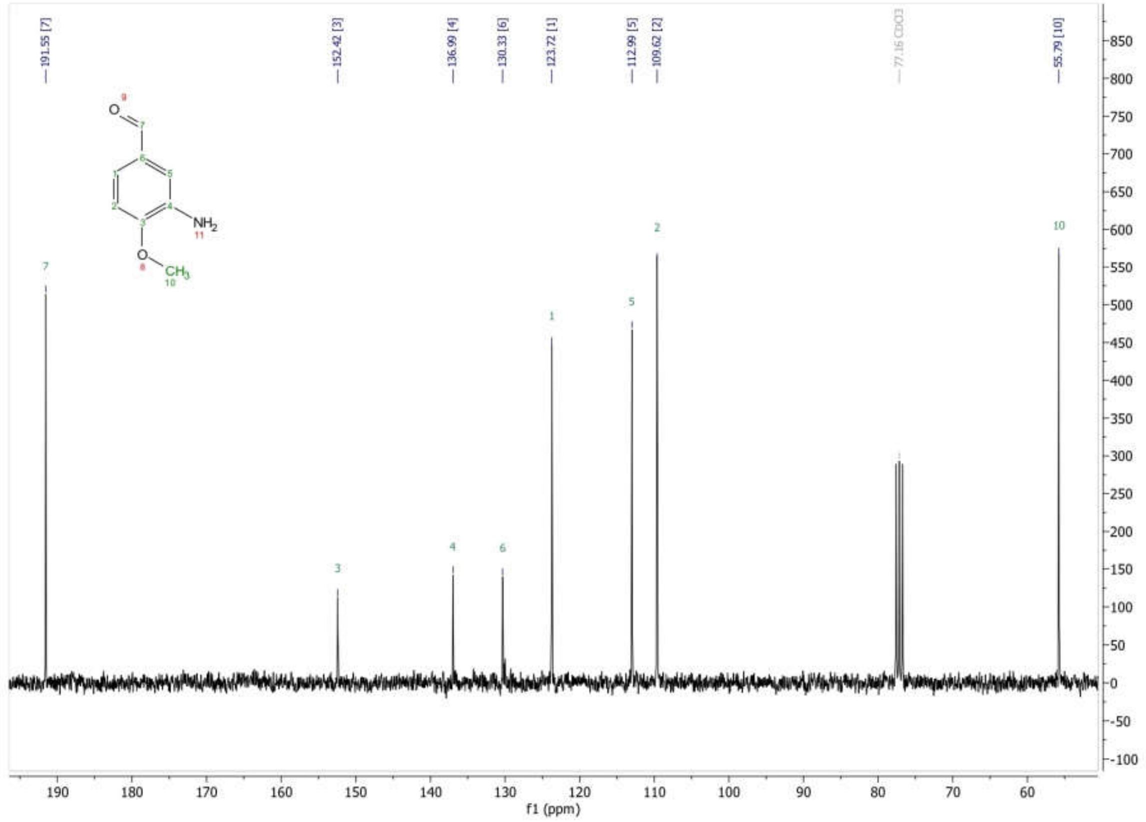

^{1}H- and ^{13}C-NMR spectra of compound **7**

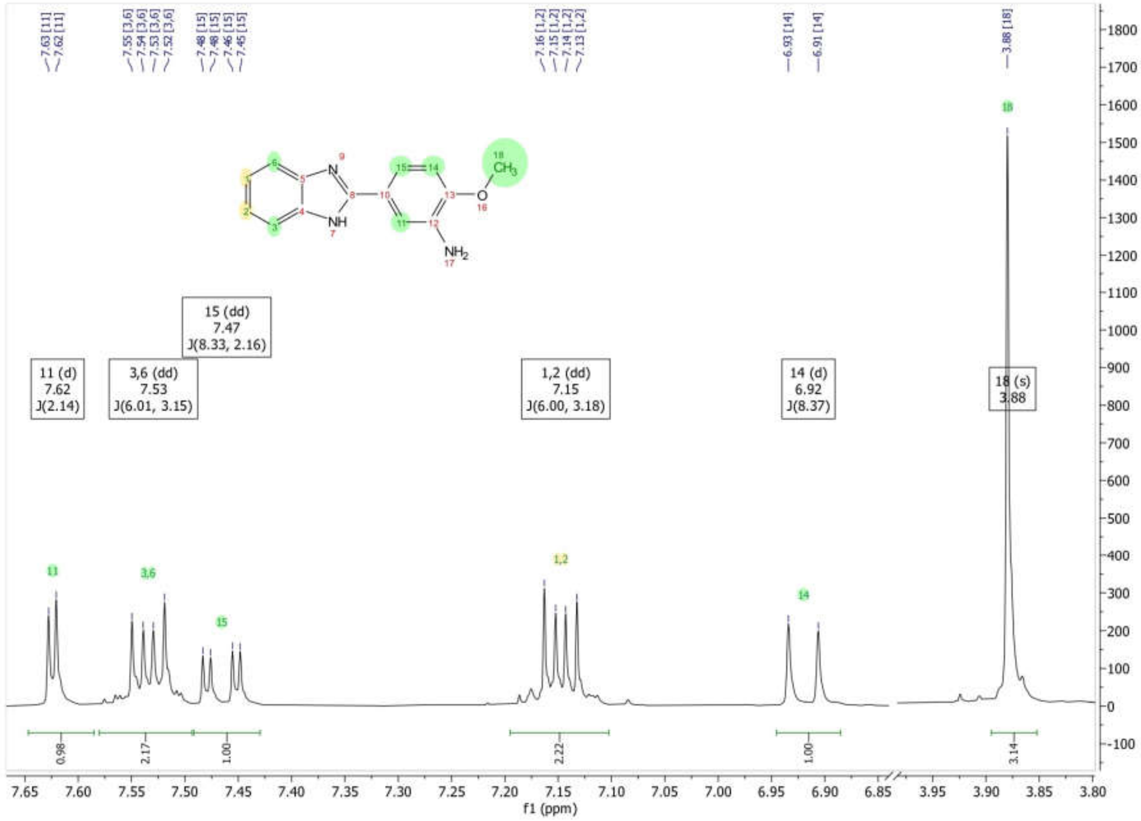

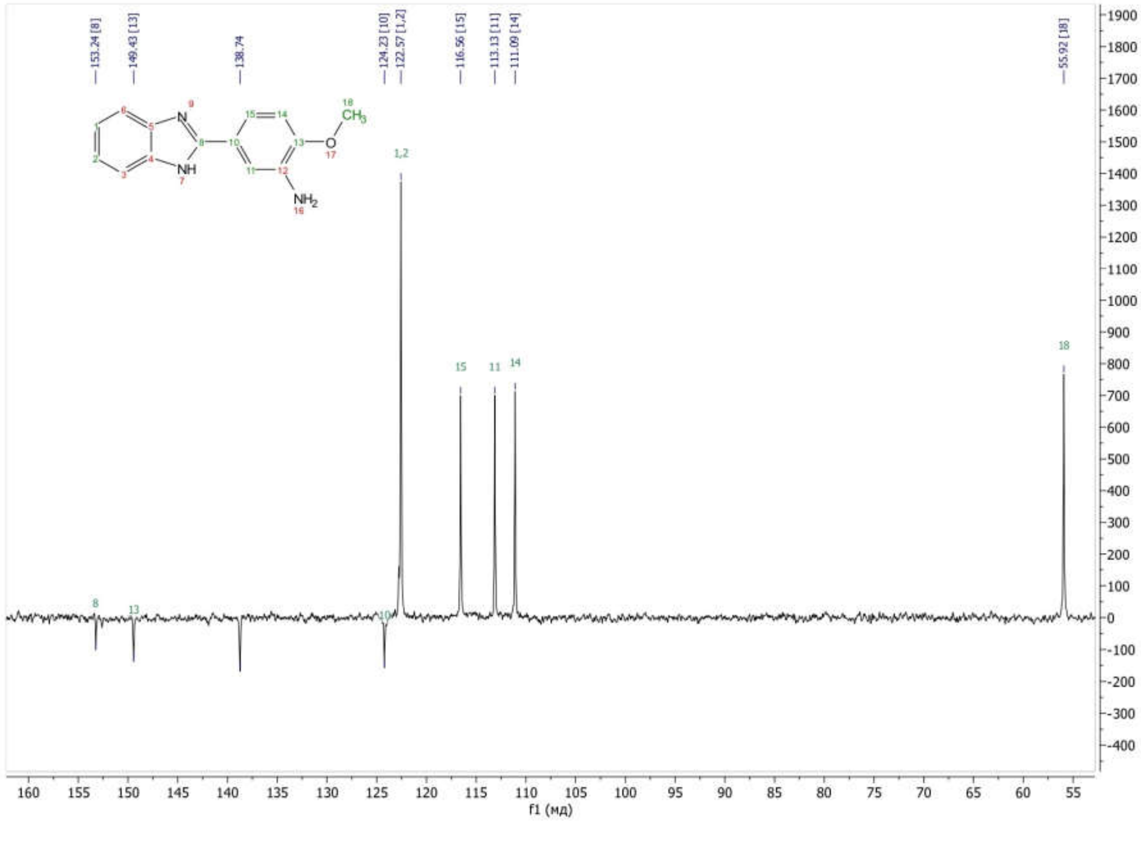

^{1}H- and ^{13}C-NMR spectra of compound **8**

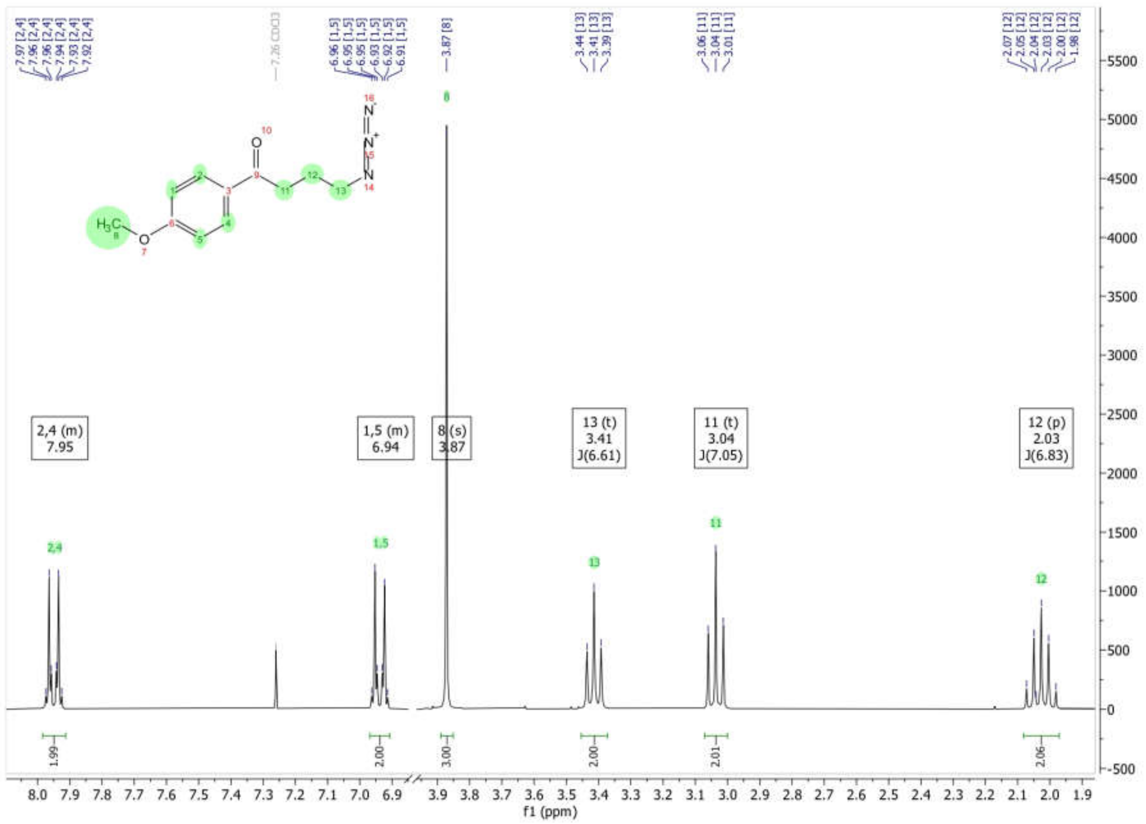

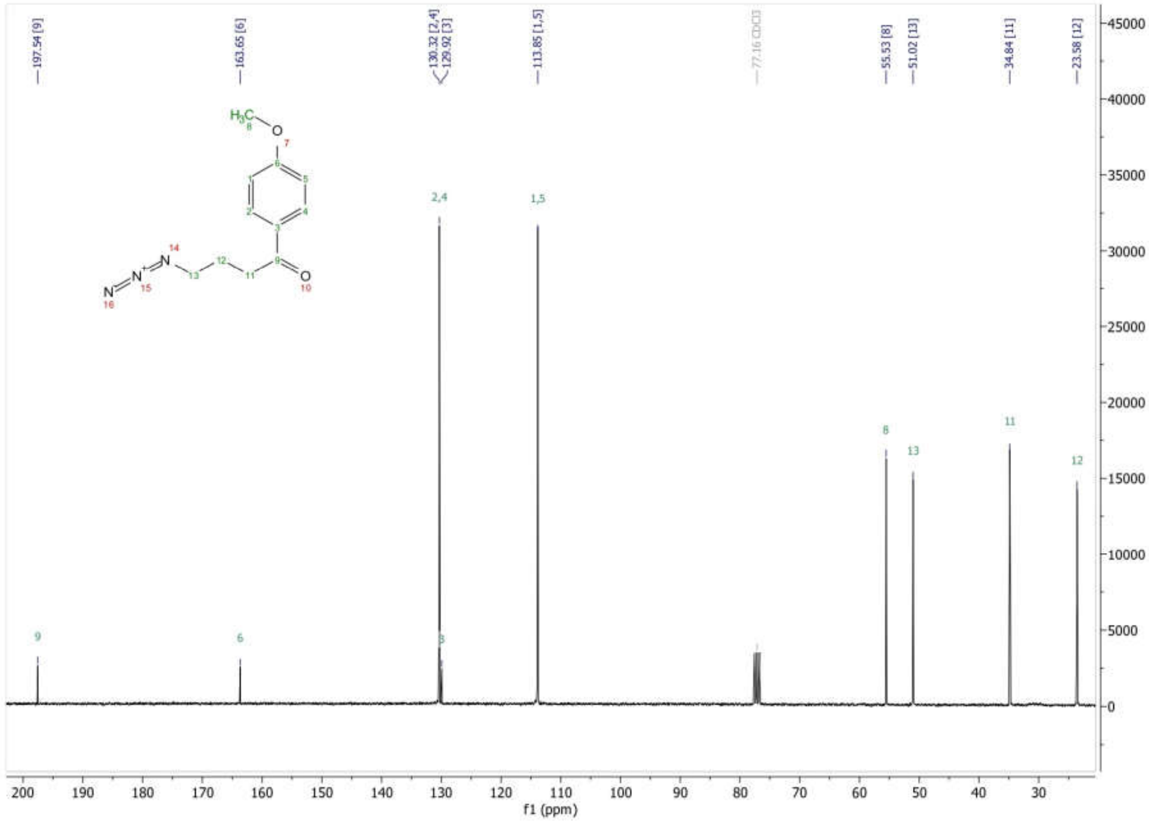

^{1}H- and ^{13}C-NMR spectra of compound **9**

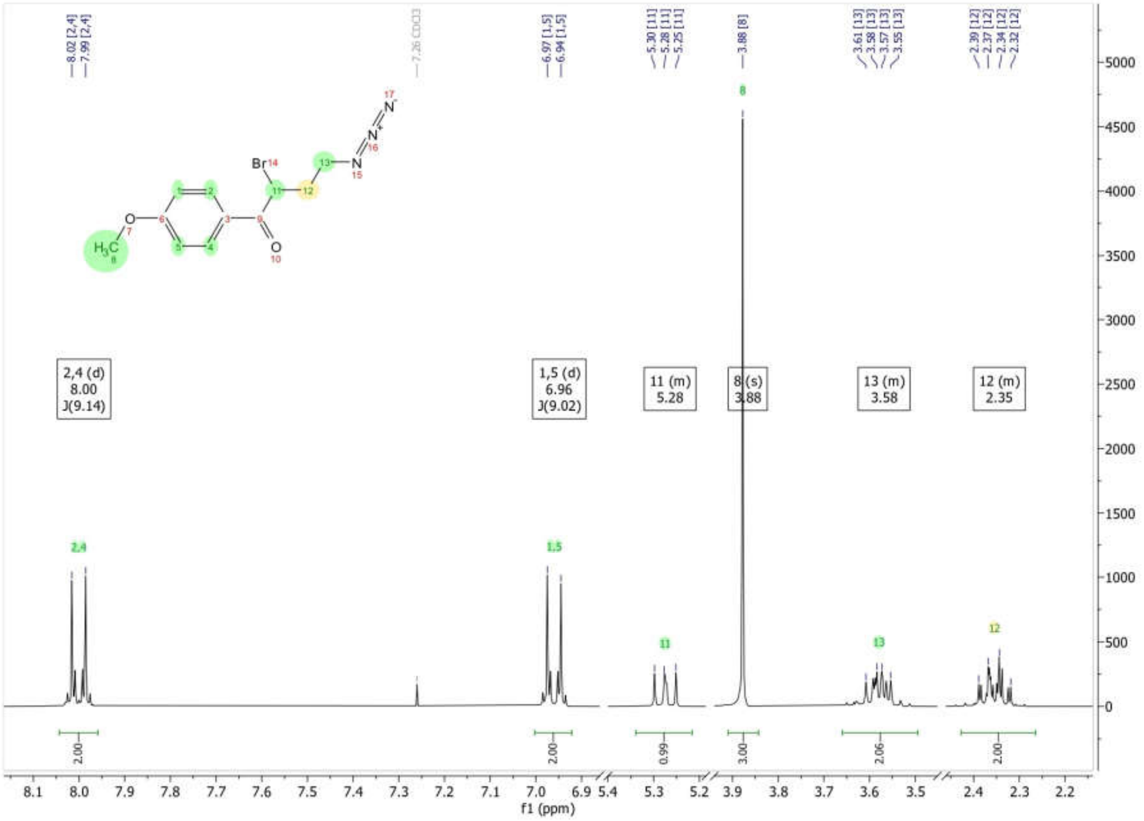

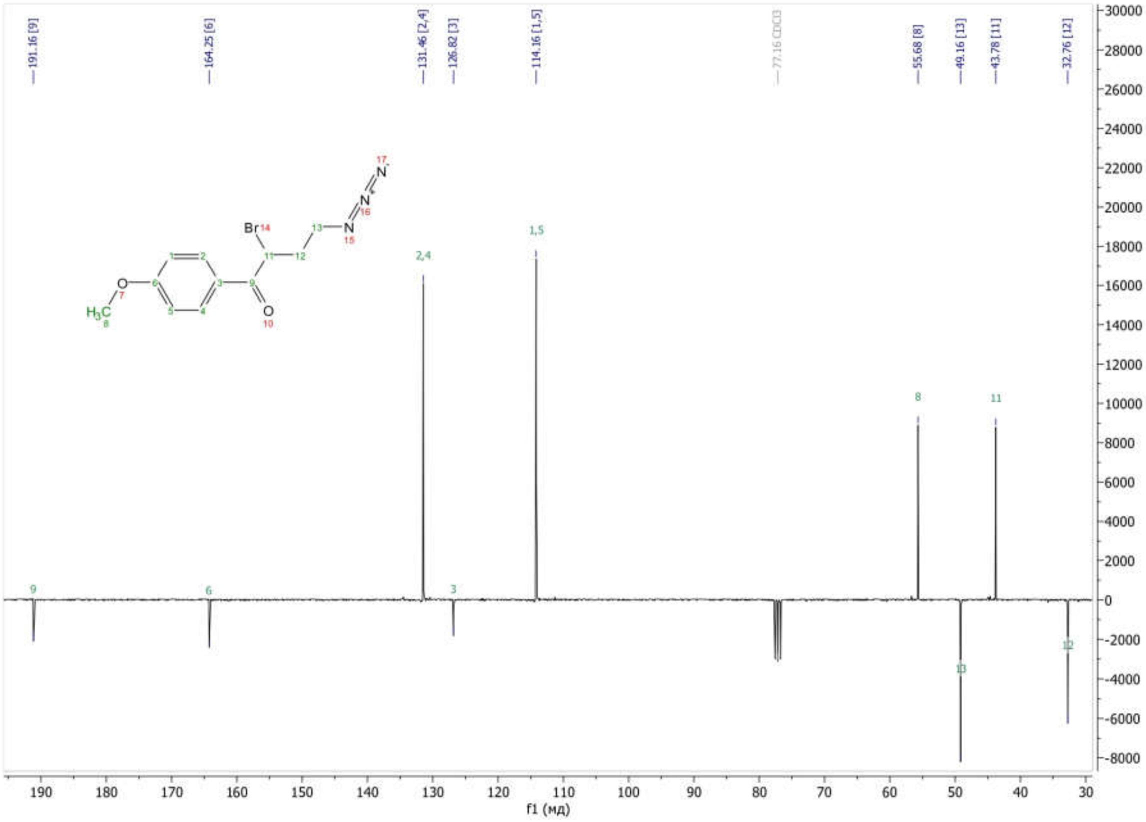

^{1}H- and ^{13}C-NMR spectra of compound **10**

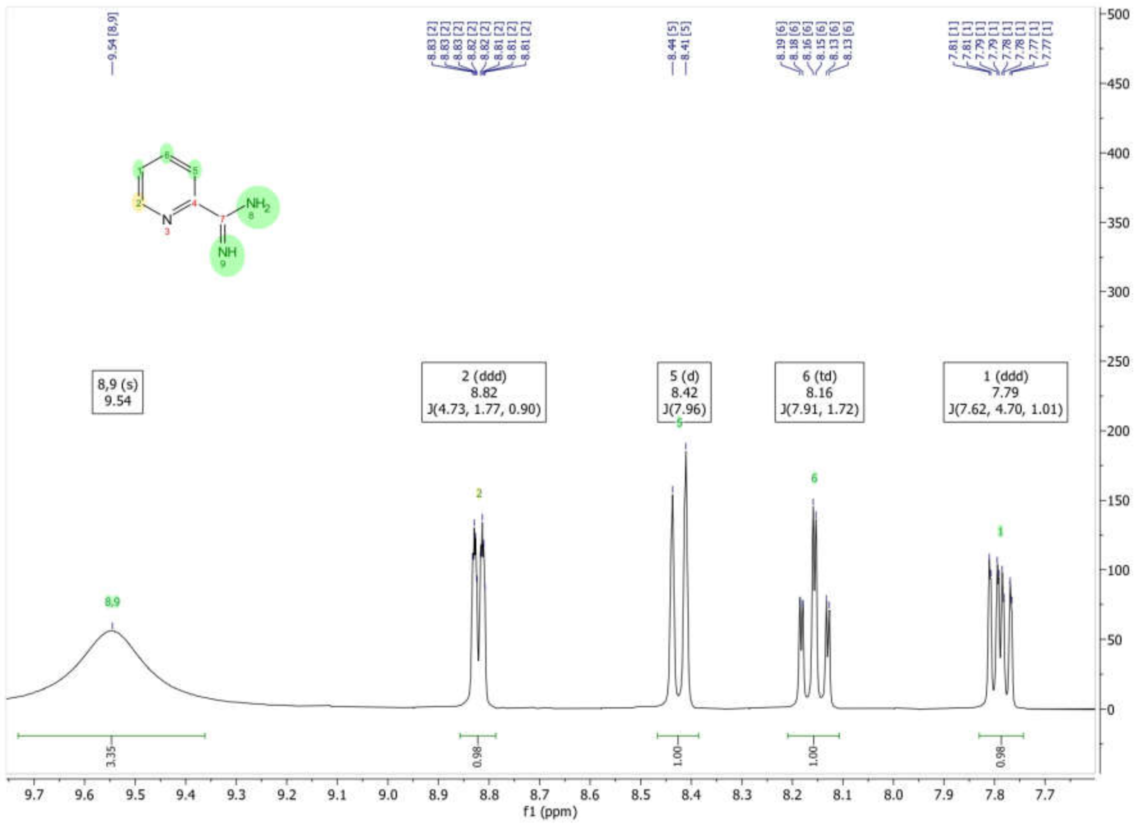

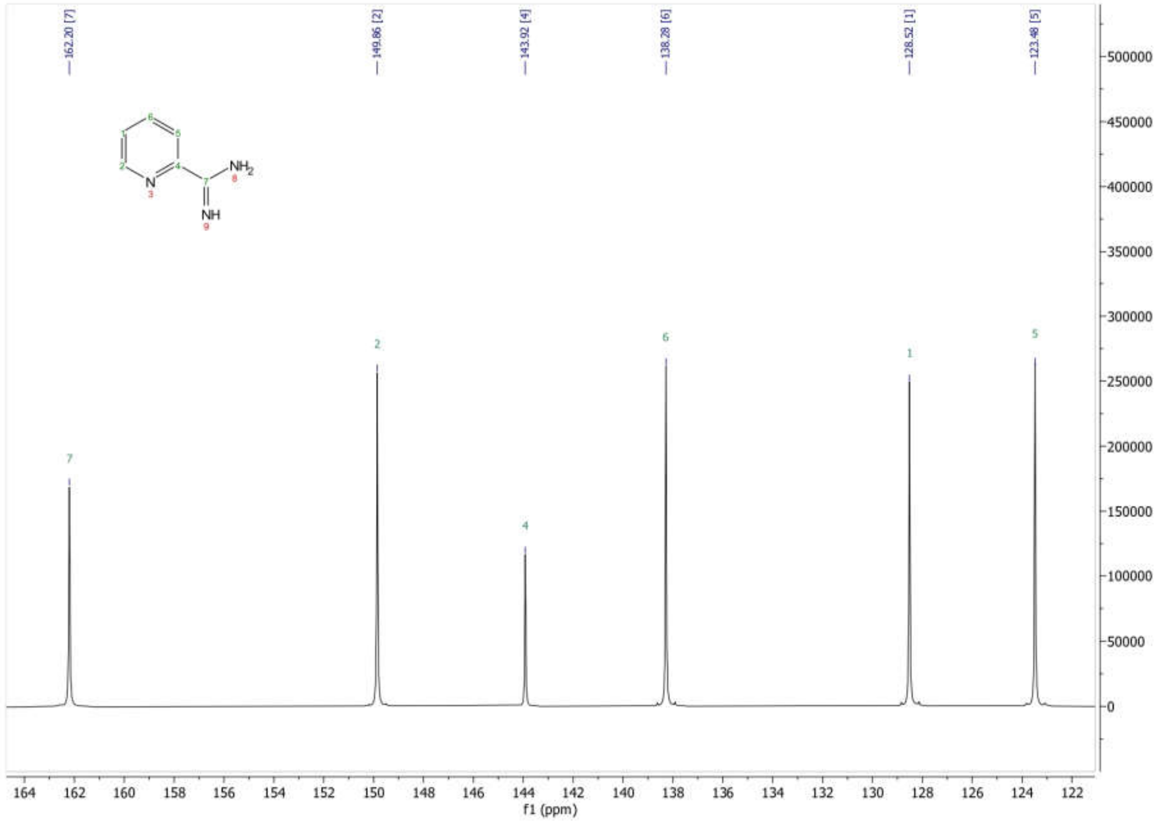

^{1}**H- and** ^{13}**C-**NMR spectra of compound **13**

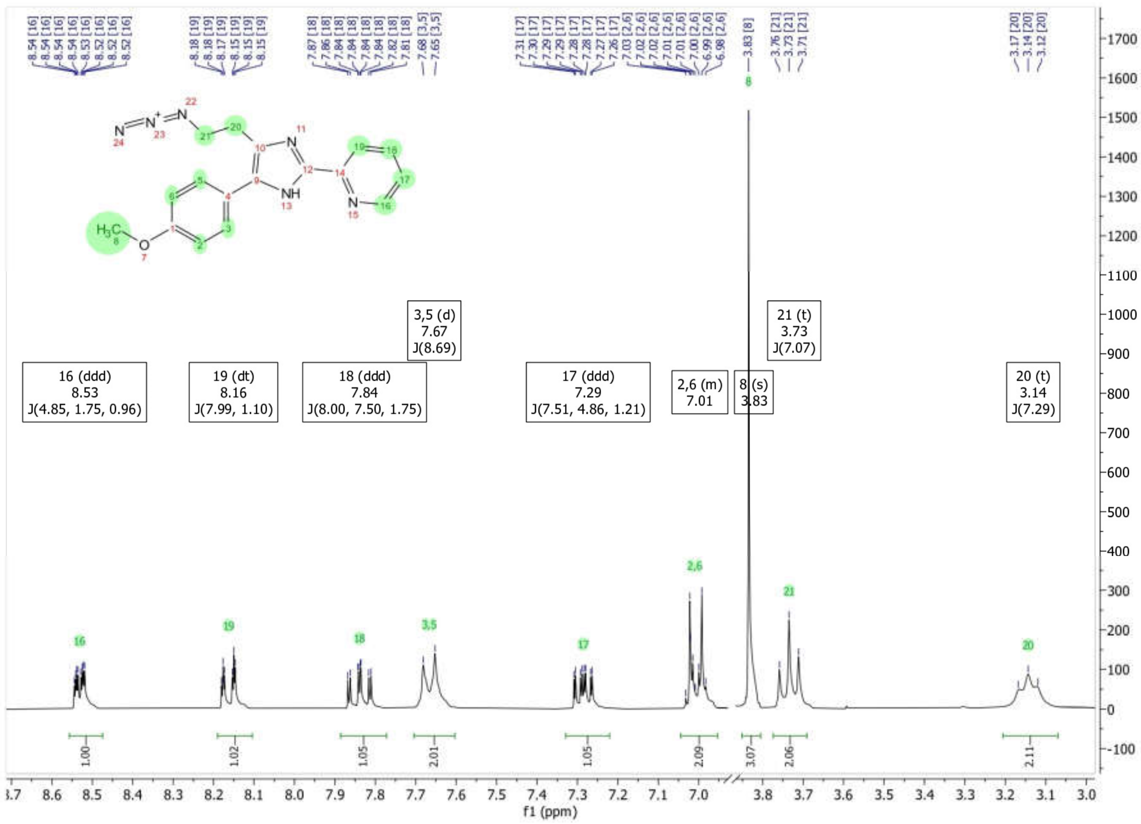

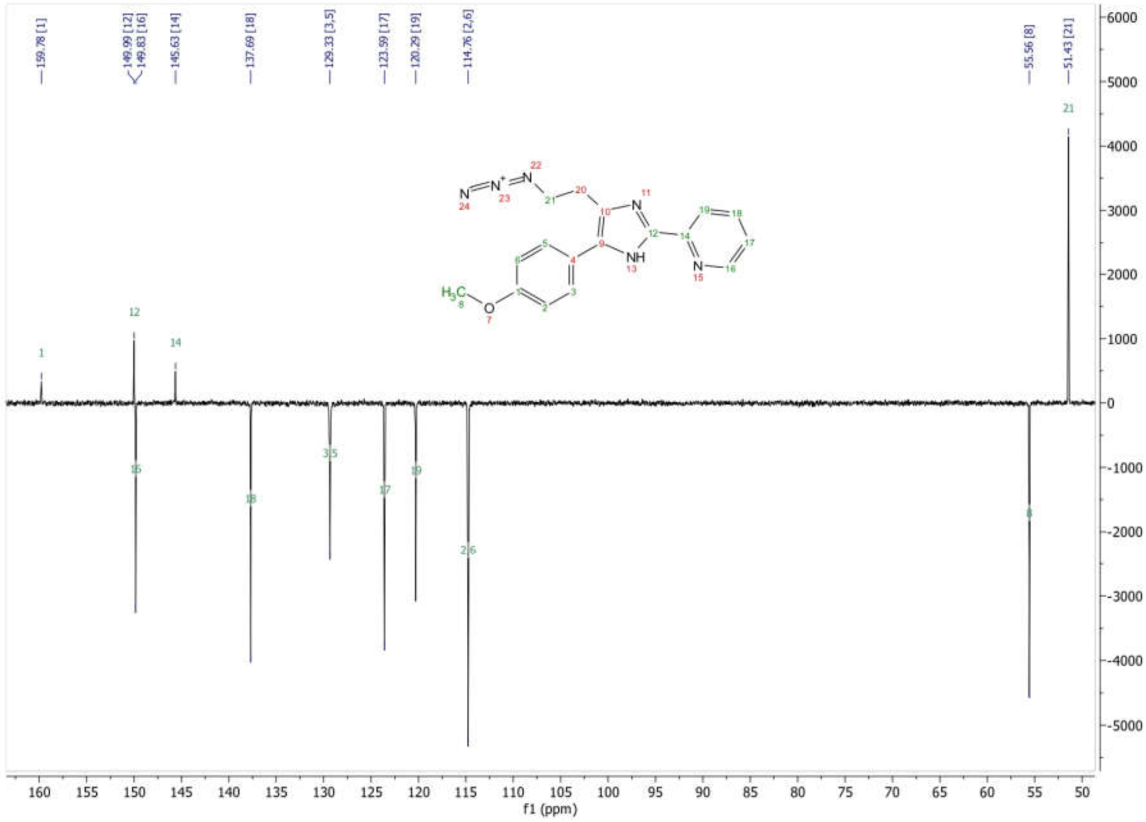

^{1}H- and ^{13}C-NMR spectra of compound **14**

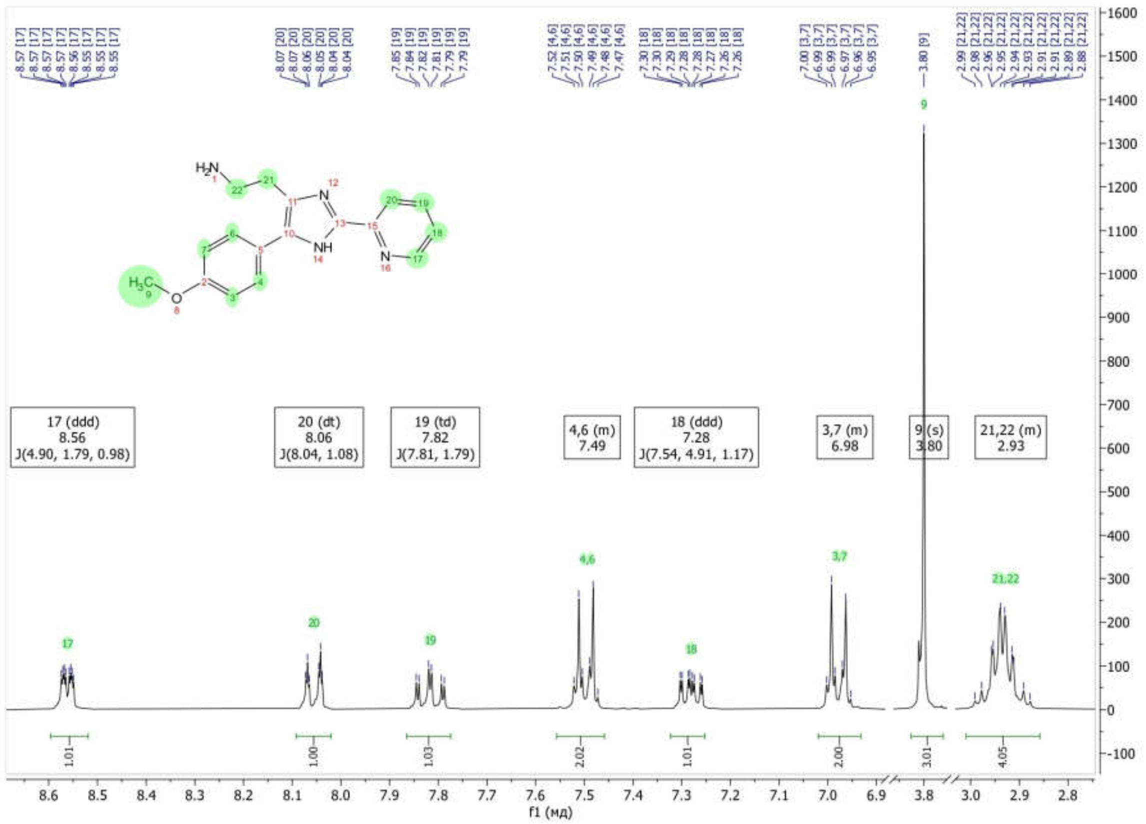

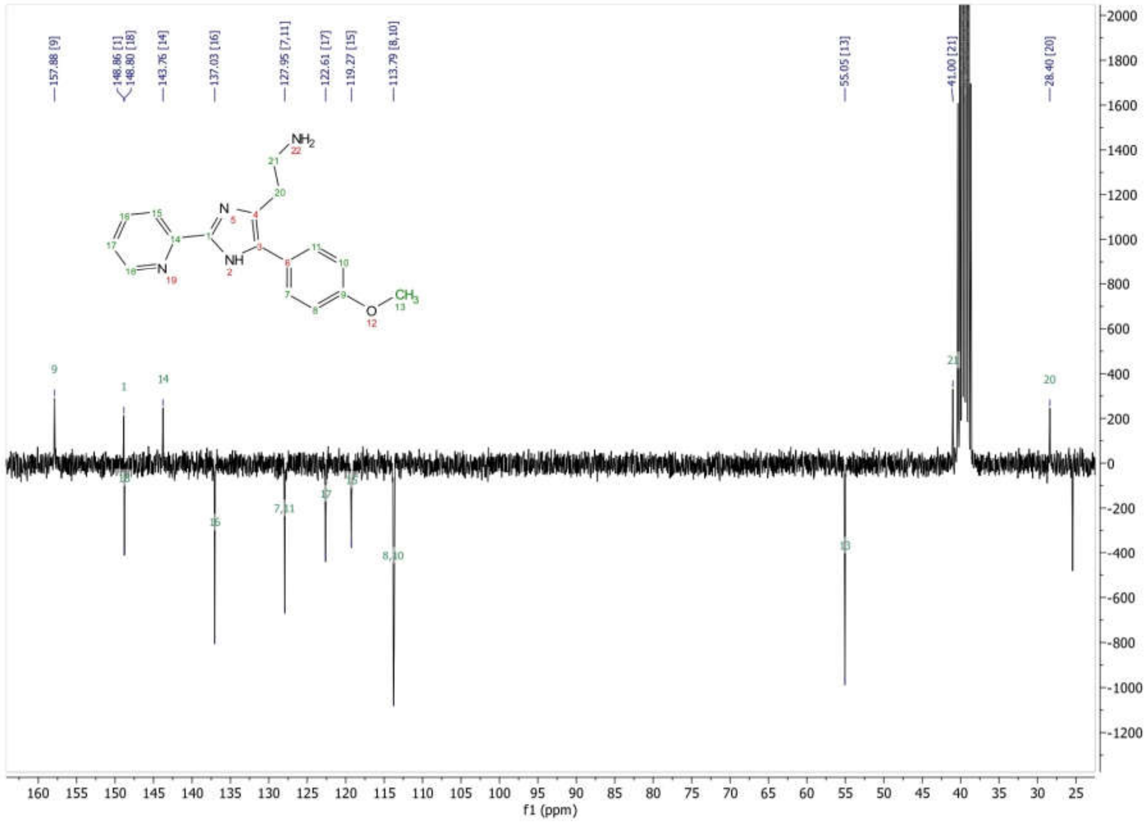

^{1}H- and ^{13}C-NMR spectra of compound **15**

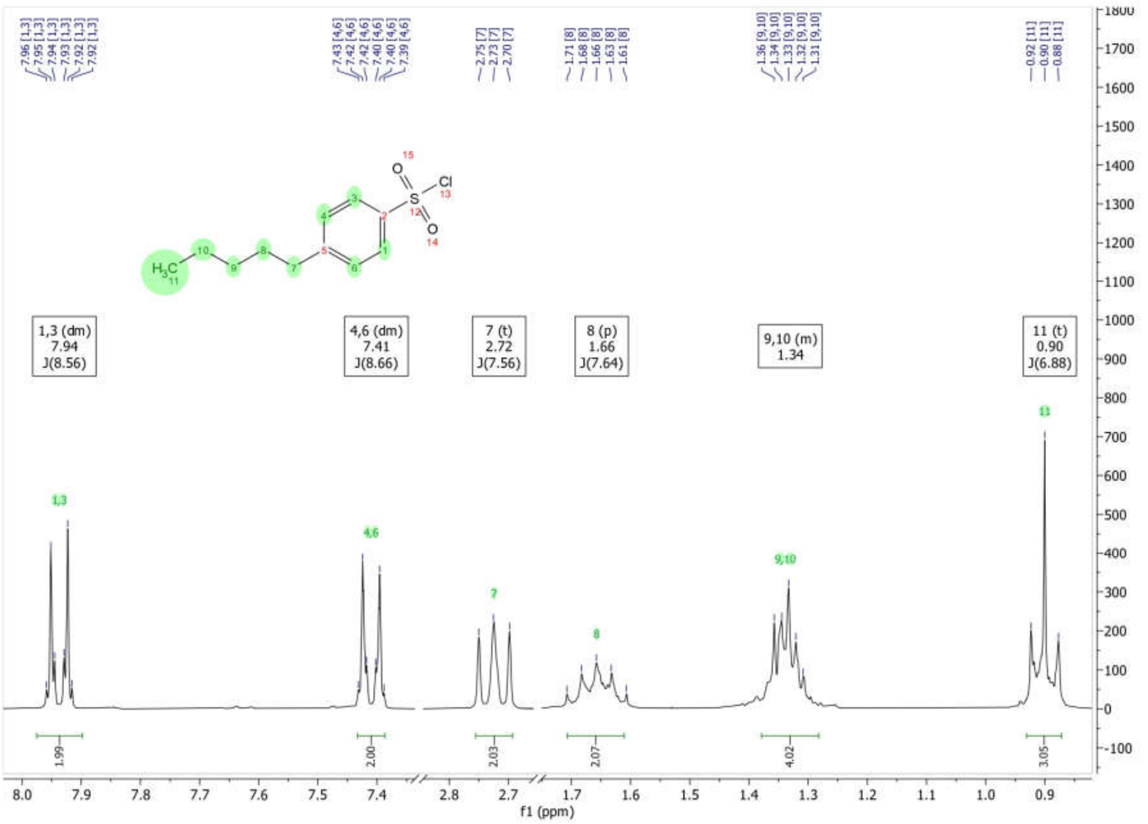

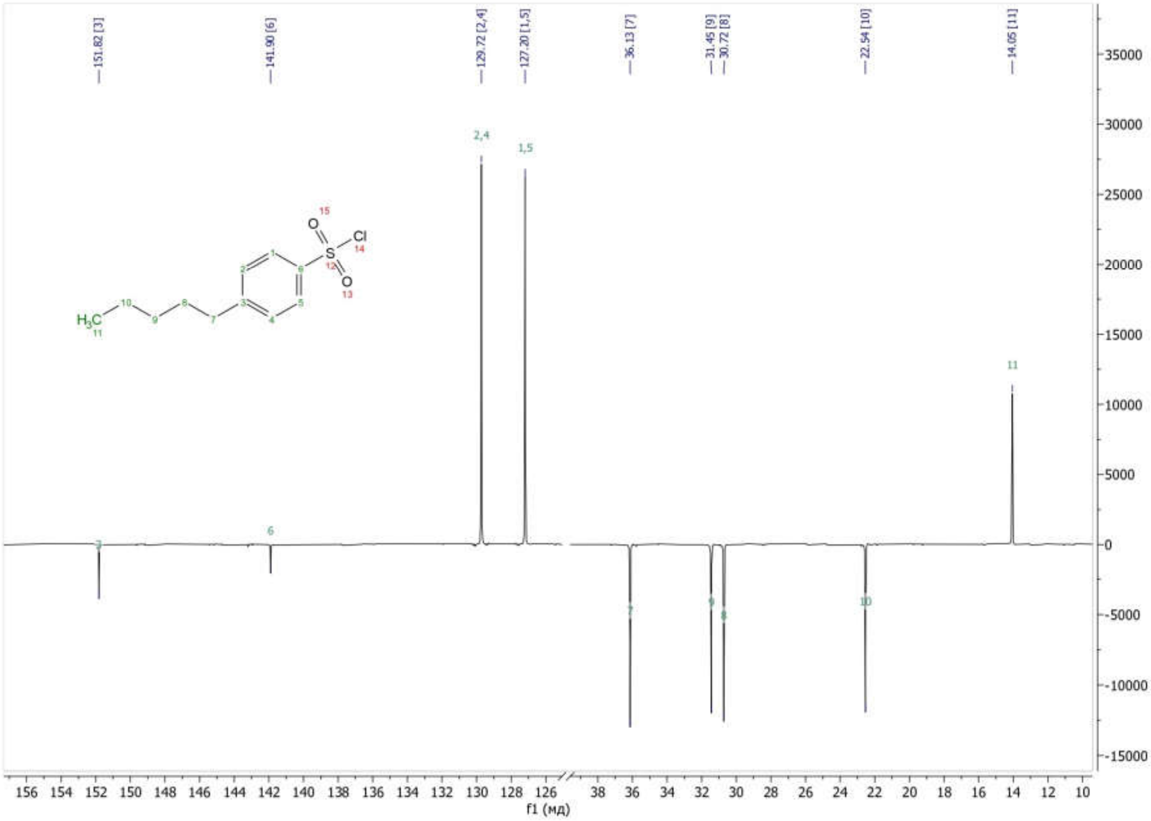

^{1}H- and ^{13}C-NMR spectra of compound **17**

3.2. MS spectral analysis

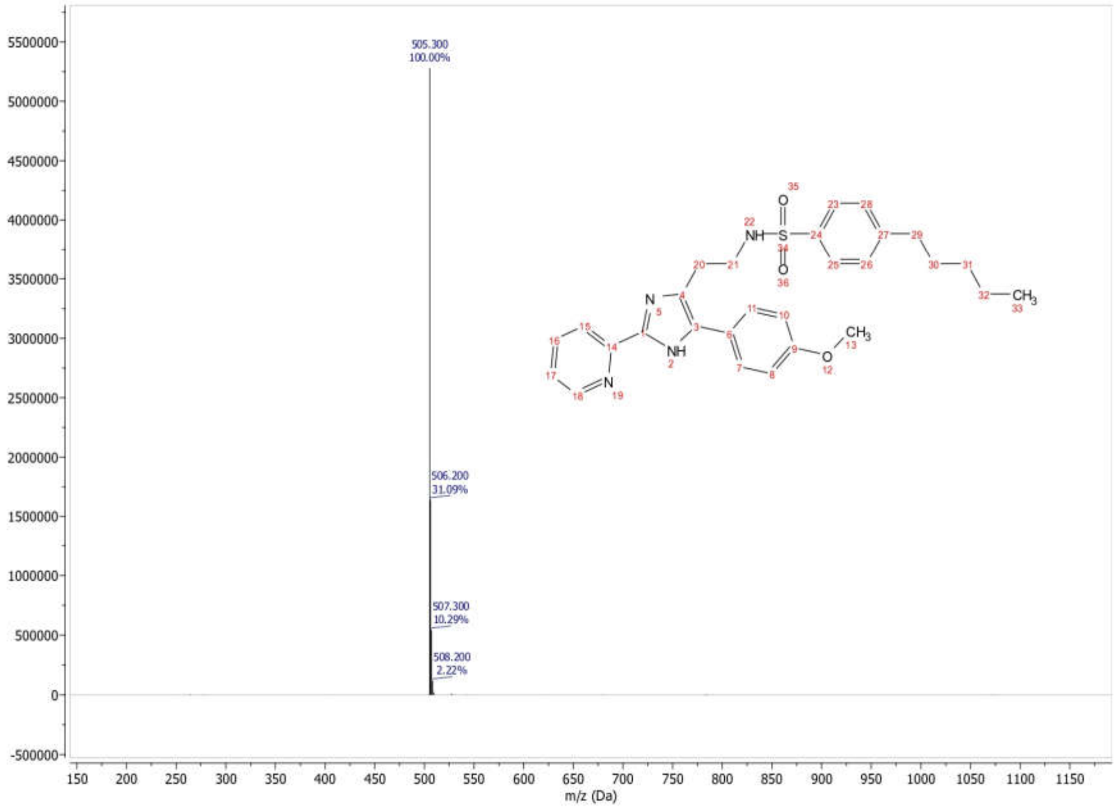

Mass-spectrum ESI of compound **2**

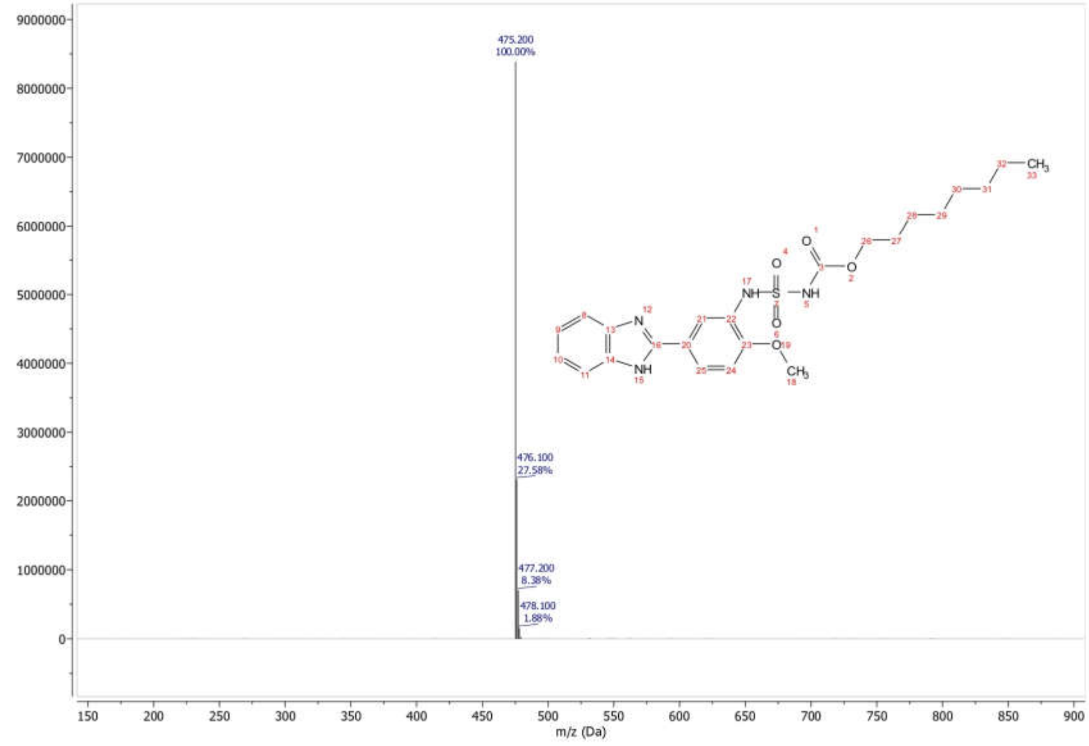

Mass-spectrum ESI of compound **3**

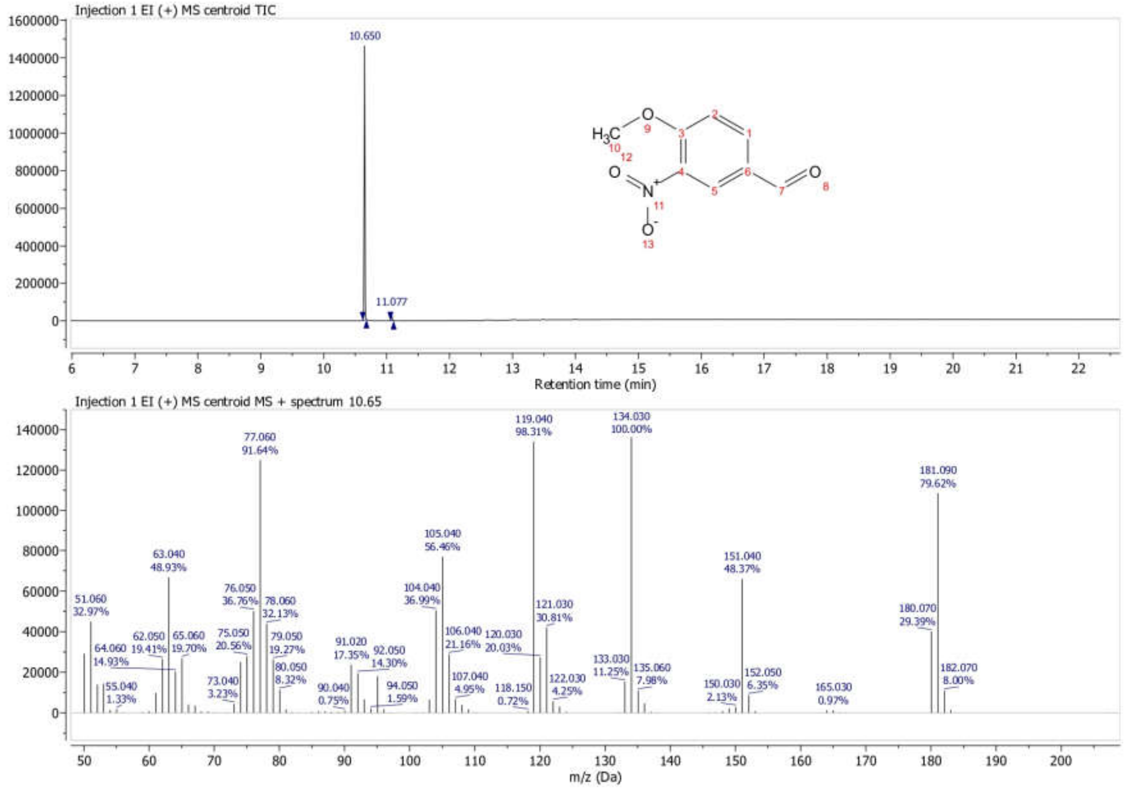

TIC and mass-spectrum of compound **6** (EI, 70 eV)

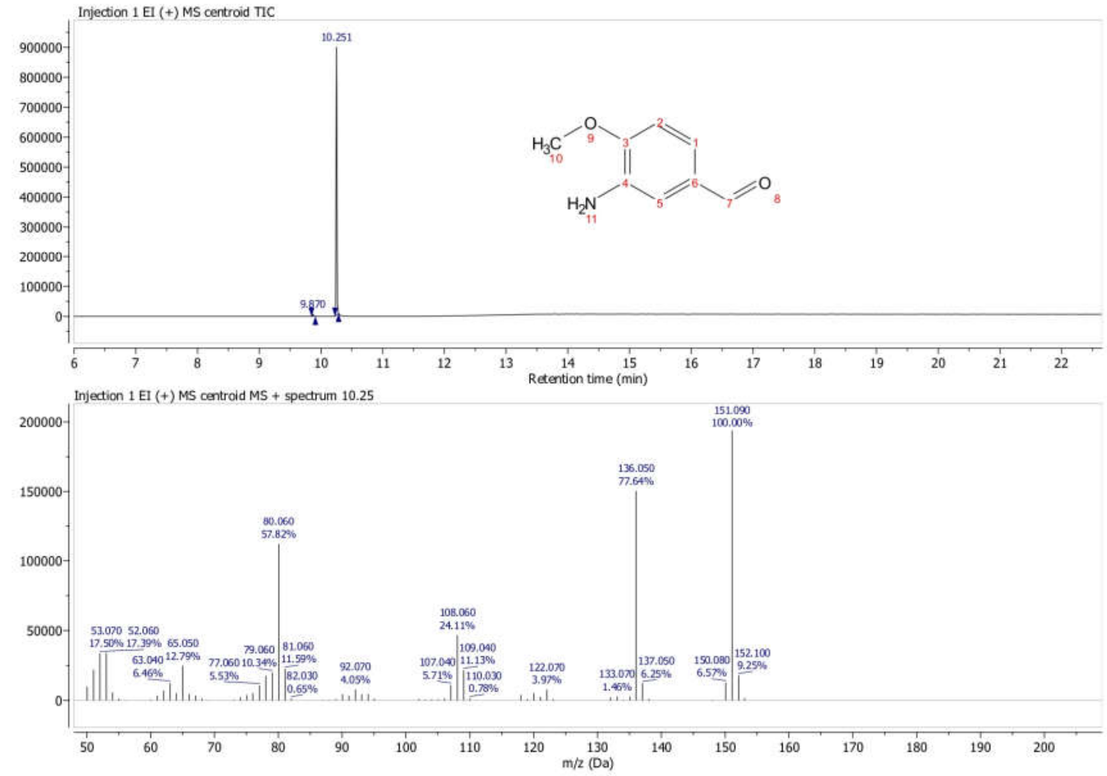

TIC and mass-spectrum of compound 7 **(EI, 70 eV)**

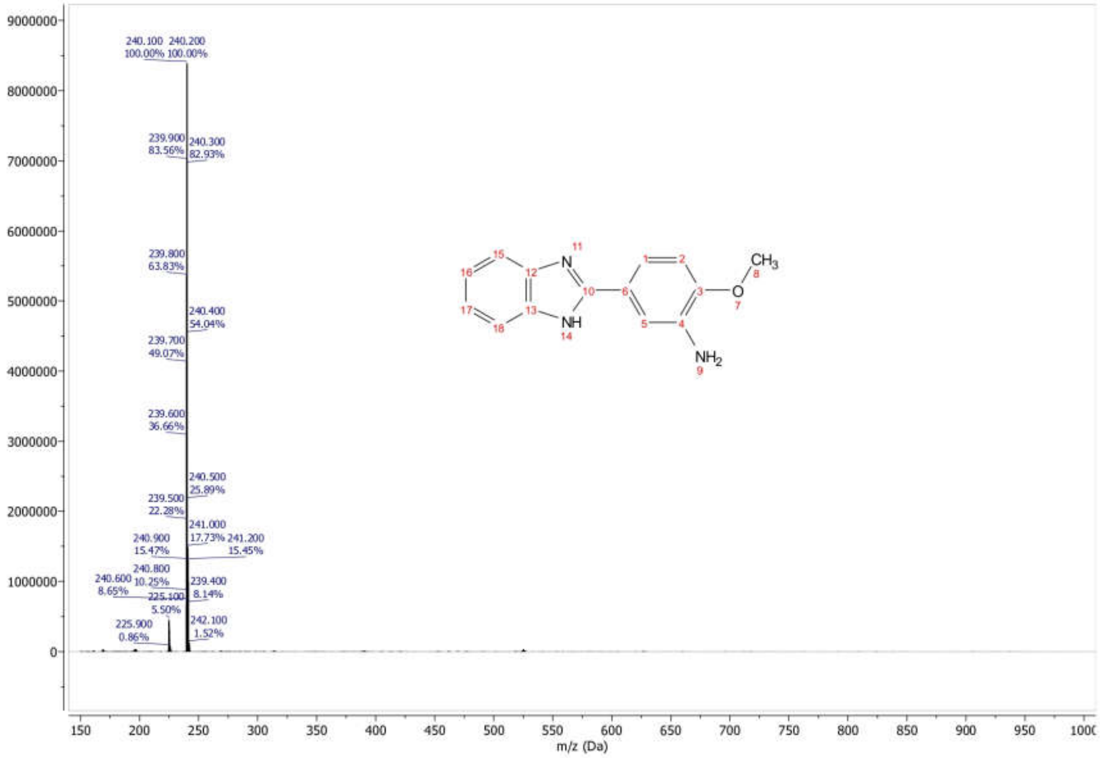

Mass-spectrum ESI of compound **8**
